# De novo DNA-based catch bonds

**DOI:** 10.1038/s41557-024-01571-4

**Published:** 2024-06-24

**Authors:** Martijn van Galen, Annemarie Bok, Taieesa Peshkovsky, Jasper van der Gucht, Bauke Albada, Joris Sprakel

**Affiliations:** 1 Laboratory of Biochemistry, Wageningen University & Research, Wageningen, Netherlands; 2 Physical Chemistry and Soft Matter, Wageningen University & Research, Wageningen, Netherlands; 3 Laboratory of Organic Chemistry, Wageningen University & Research, Wageningen, Netherlands

**Keywords:** Physical chemistry, Supramolecular chemistry, DNA nanotechnology, Biochemistry

## Abstract

All primary chemical interactions weaken under mechanical stress, which imposes fundamental mechanical limits on the materials constructed from them. Biological materials combine plasticity with strength, for which nature has evolved a unique solution—catch bonds, supramolecular interactions that strengthen under tension. Biological catch bonds use force-gated conformational switches to convert weak bonds into strong ones. So far, catch bonds remain exclusive to nature, leaving their potential as mechanoadaptive elements in synthetic systems untapped. Here we report the design and realization of artificial catch bonds. Starting from a minimal set of thermodynamic design requirements, we created a molecular motif capable of catch bonding. It consists of a DNA duplex featuring a cryptic domain that unfolds under tension to strengthen the interaction. We show that these catch bonds recreate force-enhanced rolling adhesion, a hallmark feature of biological catch bonds in bacteria and leukocytes. This Article introduces catch bonds into the synthetic domain, and could lead to the creation of artificial catch-bonded materials.

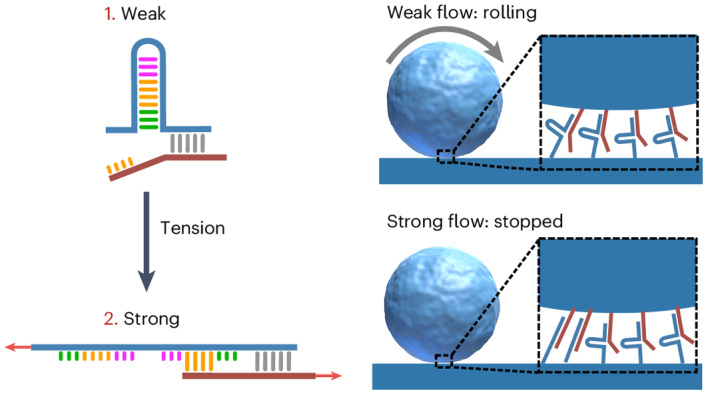

## Abstract (web summary)

Whereas bonds that strengthen under mechanical stress occur frequently in nature, all synthetic bonds weaken under force. Now, an artificial supramolecular catch bond has been shown to strengthen the particle–surface interface under increasing shear flow in a rolling adhesion assay, thereby mimicking biological adhesion processes.

## Main

Mechanical stress accelerates the dissociation of all known primary chemical bonds, both covalent and supramolecular^1–4^. This so-called ‘slip bond’ nature of chemical interactions has profound effects on the mechanical stability of molecules and their materials. In particular, bond strength and dissociation dynamics are intrinsically coupled—the stronger the bonds in a material, the slower their exchange dynamics will be. As a result, for slip bonds, supramolecular dynamics and structural plasticity are at odds with toughness and mechanical strength.

**Fig. 1:**
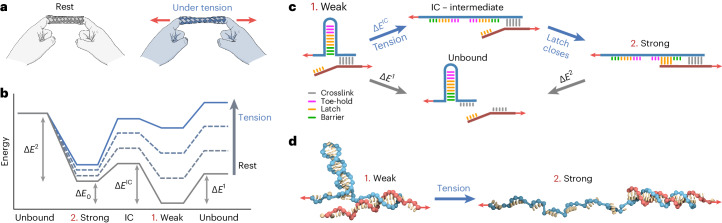
Conceptual design of a de novo DNA catch bond. **a**, Schematic of a fingertrap toy that tightens under tension, forming a stronger interaction. **b**, Conceptual energy landscape of a two-state, two-pathway catch bond. The activation barriers of transitions are indicated as *E*^*x*^. At rest, catch bonds dissociate quickly from weak state 1. Tension 'tilts' the energy landscape, trapping the catch bond in strong state 2, which dissociates more slowly. **c**, Mechanism of the de novo DNA catch bond. Weak state 1 dissociates quickly, via breaking of the crosslink. Tension opens the DNA hairpin in the hairpin strand, forming an intermediate state. When the latch closes, the catch bond transitions to strong state 2, from which dissociation is slower. **d**, oxDNA simulation snapshots of a catch bond switching from weak state 1 to strong state 2 under tension.

In nature, cells continuously experience large mechanical stresses and deformations. Cellular materials must exhibit the strength and toughness to withstand these mechanics while simultaneously remaining plastic and adaptive to accommodate the biological processes in which they are involved. Nature has evolved protein–protein and protein–ligand interactions with the unique, counterintuitive feature of strengthening under tension^5–10^. Catch bonding often results from a force-gated conformational switch in a binding pocket, converting a weak interaction into a mechanically stronger one as the tensile force on the bond increases^9,11–14^. Catch bonds are molecular equivalents of the fingertrap toy (Fig. 1a), which tightens its grip under increasing tension—the harder one tries to escape, the more difficult it becomes^12,15,16^.

Despite being a recent discovery, first hypothesized in 1988^15^ and confirmed experimentally in the early 2000s^17,18^, catch bonds are emerging as crucial design elements in nature’s mechanical materials. Catch bonds have been identified in cell-adhesion complexes^16,18–21^, cytoskeletal crosslinkers^5,8,9,22^, protein–DNA bonds involved in cell division^10,23^ and adhesive interfaces between plasma proteins and blood platelets during blood clotting^24,25^. Catch bonds play an important role in mechanotransduction^26–28^ and are proposed to allow nature’s materials to combine structural plasticity and adaptivity in quiescent conditions, while being strong and tough under loading, when needed to protect cellular integrity^9,29–31^. So far, catch bonds are only found in nature. Several theoretical models for the creation of artificial catch bonds have been proposed in recent years^32–35^. These rely on a variety of mechanisms, including force-activated hinge-based switches^33,34^, self-interacting polymer chains inspired by the catch bond von Willebrand factor and load-sharing between tethered nanoparticles^35^. Although these models provide interesting concepts, none has led to experimental realizations as yet. As a result, the promise of catch bonds as mechanoadaptive building blocks for the creation of biomimetic materials that combine plasticity with mechanical strength remains untapped.

In this Article we report the design and realization of de novo catch bonds, constructed from engineered DNA duplexes. Based on a minimal thermodynamic model for catch bonds, we design DNA duplexes that exhibit catch bonding. These duplexes contain a cryptic domain that is stable in the absence of force, but rapidly destabilizes under tension to expose additional base pairs that strengthen the bond mechanically. To characterize these designs, we take inspiration from nature. A prototypical feature of cell-adhesion catch bonds, for example, in leukocytes, is that they impart cells with the capacity to roll along tissue surfaces and become arrested at sites of high shear stresses^36,37^. We show, by conducting rolling adhesion experiments^36,38,39^ on microparticles decorated with our de novo catch bonds, that these designs can recreate this behaviour in a purely synthetic system. These experiments conclusively show how the catch bonds enhance adhesive interactions under increasing load, while slip-bond control designs show the exact opposite.

## Results and discussion

Biological catch bonds, based on protein–protein or protein–ligand interactions, are incredibly complex, and the molecular mechanism underlying catch bonding has only been conclusively evidenced for several examples^12–14,21,40^. To design a de novo catch bond, we started by abstracting the complexity of biological catch bonds to its bare essentials in the form of a set of minimal thermodynamic design criteria.

We started from the established two-state/two-pathway model^19,41,42^, in which a bond can exist in two states: a thermodynamically favoured but mechanically weak state (1. Weak) and a thermodynamically less favoured but mechanically strong state (2. Strong). Both states can reversibly convert into each other, or rupture, also reversibly, to an unbound state. This can be schematically depicted as the energy landscape of a supramolecular reaction network (Fig. 1b). Within the Kramers–Bell picture of mechanically enhanced bond dissociation, tensile forces *F* on the bonds reduce the activation barriers *E* for the transitions in this landscape, and thus the rates by which these barriers are crossed^1^. At rest, the weak state (1) is preferred. Under tension, however, the energy landscape tilts by the mechanical work supplied to the bond, and this results in a conformational switch that accesses the strong state (2), which dissociates more slowly.

To operate according to this mechanism, our catch bond should follow four thermodynamic design requirements. First, we require that state 2 is has greater mechanical strength than state 1, which is the case when *E*^2^ > *E*^1^ (requirement I). The weak state can then either rupture, with barrier *E*^1^, or convert into the strong state, with barrier *E*^IC^. In the absence of tension, the weak state should rupture and not convert to the strong state: that is, *E*^IC^ > *E*^1^ at low tension (requirement II). By contrast, under tension, the opposite must happen—conversion to the strong state happens more readily than bond rupture, such that *E*^IC^ < *E*^1^ at high tension (requirement III). Finally, we require that the thermodynamically preferred state in the absence of tension is the weak state, meaning that Δ*E*_0_ > 0 (requirement IV).

We used DNA duplexes to design a catch bond that meets these four requirements (Fig. 1c; Supplementary Table 1 presents the sequences). DNA interactions are ideal building blocks for nanotechnological structures with tailored mechanical properties, as has been demonstrated in constructs such as DNA force sensors^43,44^ and artificial motor systems^45,46^. Double-stranded DNA is remarkably elastic at low forces^47,48^ and offers good mechanical stability at larger forces due to *π*–*π* stacking interactions^49,50^. The large persistence length of the double helix allows for the construction of rigid DNA-origami structures^50–52^. Moreover, the programmability of DNA provides a unique tunability in the interactions.

Our design consists of two DNA oligomers, a hairpin strand (HS, blue) and a complementary strand (CS, red). The hairpin strand contains three functional domains: the latch, toe-hold and barrier domains. The latch domain is the cryptic binding site, which is complementary to a domain at the terminus of the complementary strand. In state 1, the hairpin strand and complementary strand are bound by a short DNA duplex denoted as the crosslink (grey), which dissociates quickly. When tension is applied, the hairpin unfolds, yielding an intermediate state in which the latch is exposed. On binding of the latch to its corresponding sequence on the complementary strand, the system interconverts to state 2. As this increases the number of base pairs in the crosslink section, this state is more stable than state 1, meeting requirement I. In the absence of force, the crosslink is less stable than the hairpin due to the large number of base pairs in the latter (19 base pairs) compared to the former (nine base pairs), ensuring that design requirement II is met. A mismatch of one base pair is present between the crosslink and latch sequence to separate the domains.

Design requirement III is the most challenging to achieve. It requires that the interconversion *E*^IC^ is more susceptible to tension than the dissociation barrier from state 1 (*E*^1^). We accomplish this by using stress localization based on the mechanical geometry of loading. The crosslink (barrier *E*^1^) is loaded in a shear-type configuration, where the tension is distributed evenly across all base pairs. By contrast, the hairpin (barrier *E*^IC^) is loaded in a zipper-type geometry; this localizes the tension on the first few base pairs at the start of the hairpin. This stress localization makes DNA duplexes loaded in a zip geometry mechanically unstable as compared to identical duplexes loaded in the shear geometry. This difference in mechanical stability between zip-loaded and shear-loaded DNA duplexes has been widely established in a range of studies^38,53–56^. It has been exploited previously to create DNA molecular motors relying on asymmetric binding sites that respond differently depending on the direction of the applied force^45^. Our DNA catch bond differs from this mechanism, as it strengthens under increasing force applied along a single direction. Furthermore, the difference between shear-type and zip-type unfolding pathways has been proposed previously as a mechanism for catch-bond behaviour in natural proteins too^57,58^. Requirements II and III ensure that dissociation of the weak crosslink is more likely at low tension, and opening of the hairpin followed by interconversion to the strong state is more likely at high tension. This force-dependent stability switching of the crosslink versus the hairpin lies at the core of our DNA catch-bond design.

**Extended Data Fig. 1:**
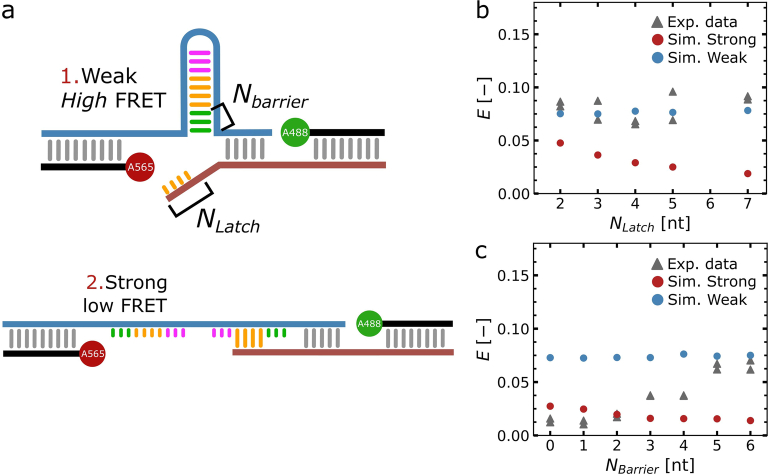
FRET spectroscopy measurements reveal that the catch bond construct assembles in the weak state 1. a: Catch bond candidates with varying latch sequence length *N*_*Latch*_ and barrier sequence length *N*_*Barrier*_ (sequences: CB-CS and CB-HS) were hybridized to Alexa Fluoro labeled DNA oligos (A488-spacer and A565-spacer). The distance between the FRET-dyes is longer in strong state 2, compared to weak state 1. This results in a lower FRET efficiency E, allowing us to distinguish the two states. b: A comparison between experimentally obtained FRET efficiencies (triangles, in duplo) and predicted FRET efficiencies using oxDNA simulations in the weak and strong states (circles) reveals that weak state 1 is the preferred state for each value of *N*_*latch*_, including the main catch bond design (*N*_*Latch*_ = 7). c: A similar comparison for FRET measurements at varying *N*_*Barrier*_ reveals that a minimum of 5 barrier nt is necessary to ensure that weak state 1 is preferred over state 2. The main catch bond design has *N*_*Barrier*_ = 6. Experimental details are provided in SI section FRET experiments on the de novo catch bond.

Finally, our design requires that in the absence of force, the construct is always fully in the weak state (design requirement IV). Through trial and error, we found that a short barrier sequence (Fig. 1c, green) is required to achieve this. This barrier sequence is designed to be complementary to the base pairs within the hairpin, but non-complementary to the corresponding poly(T) region on the complementary strand (detailed sketches are provided in Supplementary Figs. 9–20). This therefore increases the thermodynamic stability of weak state 1 relative to strong state 2. Using Förster resonance energy transfer spectroscopy and computational modelling, we confirmed that a barrier sequence of at least five base pairs in length is essential to ensure that requirement IV is fulfilled (Extended Data Fig. 1).

**Extended Data Fig. 2:**
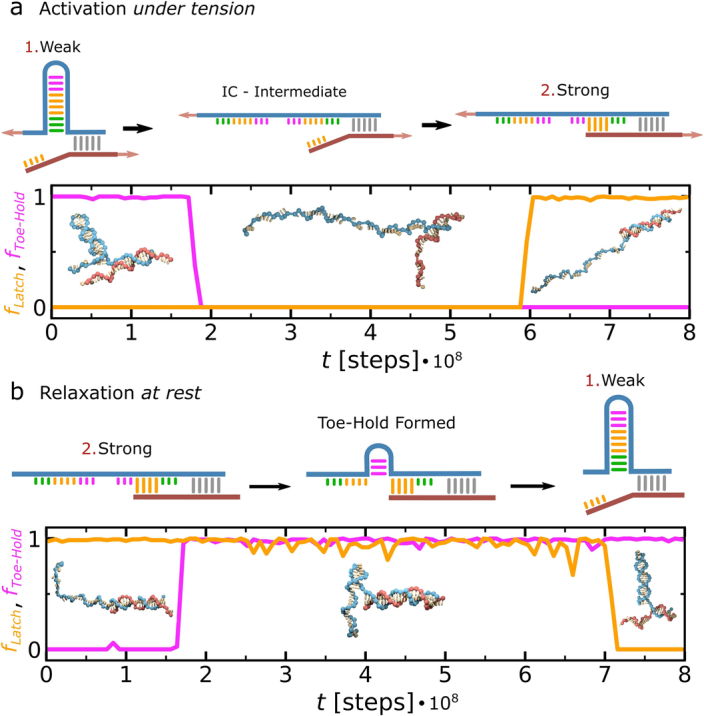
Coarse-grained molecular dynamics simulations using oxDNA show transitions between weak state 1 and strong state 2 under tension (a) and at rest (b). We track the state of the catch bond using the fraction of base-pairs formed in the toe-hold region (*f*_*toe*−*hold*_) and those formed between the complementary strand and hairpin strand in the latch region *f*_*latch*_. Simulation snapshots are shown as insets. a: When weak state 1 is placed under tension, the hairpin opens, followed by a closing of the latch to yield strong state 2. b: At rest, strong state 2 relaxes to weak state 1 via an initial formation of the toe-hold, which displaces the latch on the complementary strand. Simulation details are provided in the SI section Coarse-Grained molecular dynamics simulations.

**Extended Data Fig. 3:**
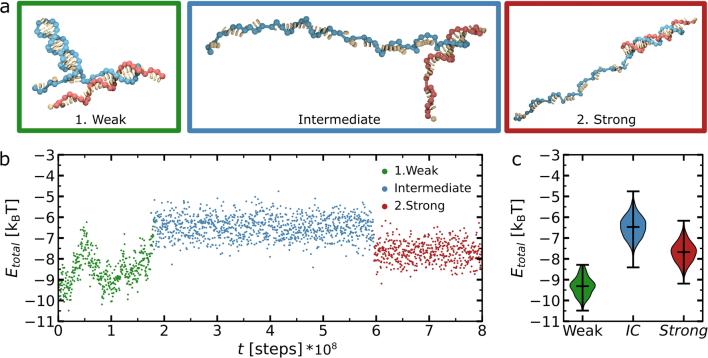
Quantification of the total energy *E*_*total*_ during the oxDNA simulation at 30 pN shown in Extended Data Fig. 2a. a: We separated the simulation in states 1, intermediate and 2 using the simulation snapshots. b: Time-evolution of *E*_*total*_ over the course of the simulation. Starting from state 1.weak, the hairpin first unfolds reversibly at *t* = 0.5 ⋯ 10^8^ steps, causing a spike in *E*_*total*_. Between *t* = 1.5 ⋯ 10^8^ and *t* = 2 ⋯ 10^8^ steps, the hairpin fully unfolds to the intermediate state (IC), which increases *E*_*total*_. Finally, a closing of the latch sequence at *t* = 6 ⋯ 10^8^ steps again lowers *E*_*total*_. c: *E*_*total*_ distributions recorded in each of the three states reveal that an energy barrier must be crossed when transitioning from state 1 via the intermediate IC to state 2. Moreover, *E*_*total*_ is lower for state 1 than for state 2, confirming that weak state 1 is the thermodynamically favored state. Here, the *E*_*total*_ distribution of state 1 represents *E*_*total*_ during the first 0.25 ⋯ 10^8^ simulation steps, prior to the reversible hairpin unfolding event at *t* = 0.5 ⋯ 10^8^. Violin plots in c are composed of N=59 (weak), N=1036 (IC), and N=512 (Strong) simulation snapshots. Error bars in c represent standard deviations. Simulation details are provided in the SI section Coarse-Grained molecular dynamics simulations.

To verify our conceptual mechanism of catch-bond activation, we used oxDNA simulations^59,60^. These show that the design indeed transitions from weak state 1 to strong state 2 under tension, via the anticipated reaction intermediate (Extended Data Fig. 2a and Supplementary Video 1). Moreover, these simulations revealed that catch-bond activation is reversible; catch bonds in state 2 spontaneously return to state 1 following release of the mechanical tension by the formation of a toe hold and internal strand exchange (Extended Data Fig. 2b and Supplementary Video 2). We estimate the typical force required for catch-bond activation to be 12−18 pN, based on previously reported oxDNA simulations on zipper-loaded DNA duplexes^61^. We further quantified the total energy *E*_total_ over the course of our catch-bond activation simulations and found that state 1 is lower in energy than state 2 (Extended Data Fig. 3). This confirms that our catch bond meets design requirement IV. We also found that the interconversion (IC) state has a larger *E*_total_ than both state 1 and state 2, suggesting that a small energy barrier must be crossed when returning from state 2 to state 1.

**Extended Data Fig. 4:**
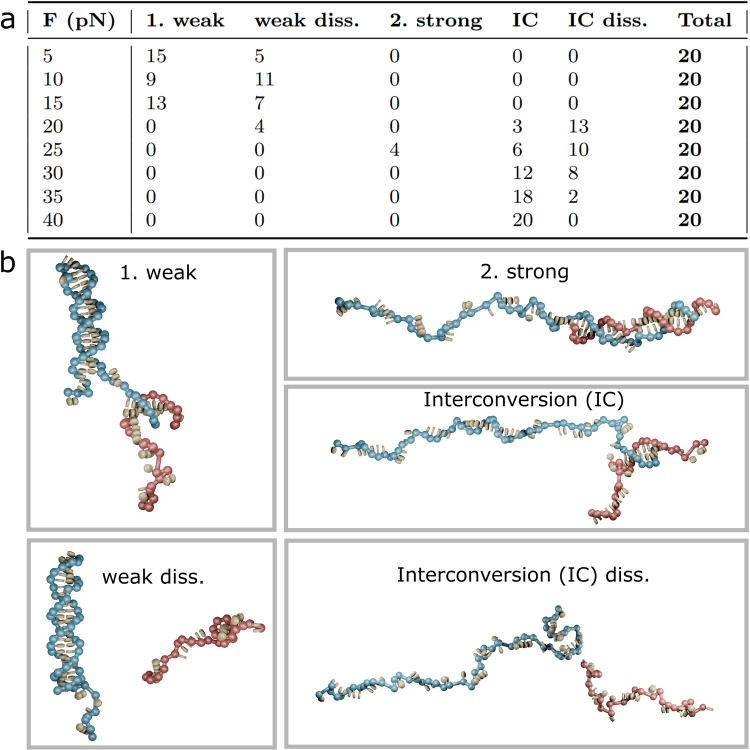
oxDNA simulations at elevated temperature reveal a changing dissociation mechanism on increasing tension. a: Overview of the number of simulations ending up in each catch bond state at varying tensional forces *F*. States include 1. weak, interconversion (IC), 2. strong and dissociation from these states. b. Simulation snapshots of each state included in a. At *F* = 15 pN the bonds are either stable in 1. weak or dissociate from 1. weak. At increasing force, the bond interconverts and in a few simulations stabilizes in state 2. strong. Larger forces result in rapid dissociation from the intermediate state before the catch bond has a chance to activate. simulations were performed at 40°C for 1.6 ⋯ 10^9^ simulation steps, with stepsize Δt = 0.005. Each simulation was repeated 20 times.

To confirm that tension changes the dissociation mechanism of the DNA catch bond, we performed an additional series of oxDNA simulations. Because the dissociation timescales are inaccessible in our oxDNA simulations at room temperature, we carried out this series at an elevated temperature (40 °C) to accelerate the dissociation timescales (Extended Data Fig. 4). At low tensional force, we often observe dissociation from the weak state of the catch bond. With increasing forces, we first observe a transition to the intermediate state, followed by a fraction of bonds that stabilize in the strong state, revealing a change in dissociation mechanism. Further increases in tensional force result in rapid dissociation of the catch bond from the intermediate state, before the latch is able to close.

To experimentally confirm the catch-bond behaviour of our DNA constructs, we made use of a rolling adhesion assay^36,39^. Cell-adhesion catch bonds, such as those on the surfaces of leukocytes, enable these cells to stabilize their adhesive interfaces under shear as they roll along surfaces that display a catch-bonding ligand^36,37,62^. Rolling—the motion of a cell without de-adhesion—occurs by the continuous exchange of a dissociating catch bond at the trailing edge with a newly formed one at the leading edge^63,64^. Rolling adhesion measurements of microparticles bound to a surface by DNA slip bonds show how increased flow (that is, increased hydrodynamic forces on the particle) promotes supramolecular kinetics and thereby enhances rolling. For catch bonds, in contrast, we expect the opposite—increased hydrodynamic forces stabilizing the adhesive contact.

**Fig. 2:**
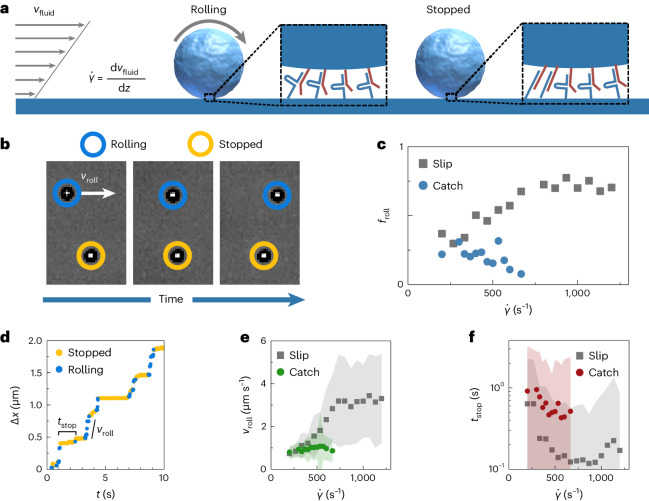
Rolling adhesion measurements reveal catch-bond behaviour. **a**, Schematic representation of a rolling adhesion experiment. Microparticles are bound to the surface of a microfluidic channel through catch bonds. A fluid flow through the channel imposes a shear rate ${\dot{\gamma }}$ across the particles, which makes them roll forward, continuously breaking and forming bonds. Stabilization of the interface, through catch-bond activation or an increase in bond density, causes particles to stop. **b**, Brightfield microscopy images showing a rolling and a stopped particle over time. **c**, Fraction of rolling particles *f*_roll_ as a function of ${\dot{\gamma }}$ for the catch-bond candidate and the slip-bond control. For catch bonds, *f*_roll_ decreases with increasing ${\dot{\gamma }}$. **d**, Displacement trace of a rolling particle, showing intermittent stop–roll behaviour, from which the stop time *t*_stop_ and rolling velocity *v*_roll_ are determined. **e**, A plot of the average *v*_roll_ for particles bound with slip and catch bonds shows that catch-bonded particles are much less sensitive to changes in ${\dot{\gamma }}$. **f**, A plot of the average *t*_stop_ over ${\dot{\gamma }}$ indicates that particles bound through catch bonds typically have larger stopping times. Shaded zones in **e** and **f** indicate standard deviations. The numbers of particle trajectories analysed per datapoint are listed in Supplementary Tables 3c and 4e,f.

**Extended Data Fig. 5:**
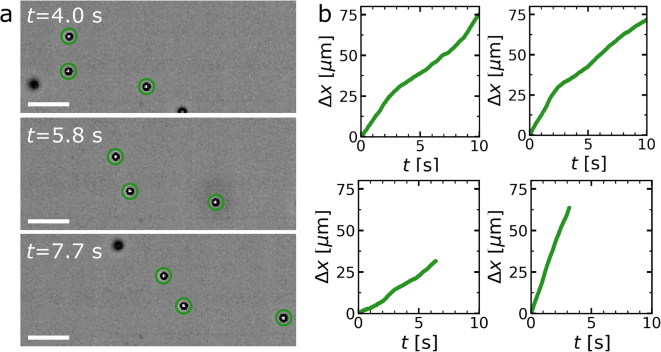
Rolling adhesion control measurement in a flow cell functionalised with 100% of the non-specific Filler DNA, at *γ*^⋅^= 16 s^−1^. a: Microparticles move rapidly across the flow cell at a shear rates as low as 16 s^−1^, more than an order of magnitude lower than the shear rates required to achieve rolling on the catch bonded or slip bonded interfaces (>200 s^−1^), confirming that any non-specific interactions in our rolling adhesion assay are much weaker than the specific slip bond and catch bond interactions. Hence, non-specific interactions do not play a substantial role in the rolling behaviour we observe for the slip and catch bonded interfaces. b: Examples of particle displacement traces recorded during these control experiments. Scale bars denote 10 *μ*m.

In our rolling adhesion assay, we applied well-defined shear rates ${\dot{\gamma }}$ across microparticles bound to the surface of a microfluidics chip using the DNA molecule of interest, placing the DNA bonds under tension (Fig. 2a). The tethered microparticles can respond in one of several ways. If the bonds in the interface reversibly break and form, we expect the particles to roll forward, continuously breaking bonds at the rear and forming new ones in front. The rolling velocity is then dictated not by hydrodynamics, but by the supramolecular reaction kinetics. If the interface is stable—for example, because catch bonds in the interface are activated—we expect the particles to become stationary (Fig. 2a). Indeed, in our experiments we observe both (Fig. 2b). We confirmed that the observed interactions are caused by specific catch and slip bonds, rather than non-specific interactions, with a control experiment in which microparticles were flowed over a flow cell surface functionalized with non-complementary filler DNA. In these measurements, particles moved rapidly across the surface at ${\dot{\gamma }}$ values more than one order of magnitude lower than those required to observe rolling on surfaces containing slip or catch bonds (Extended Data Fig. 5). This proves that any non-specific interactions present are much weaker than the specific slip and catch-bond interactions.

Through image analysis we can now compute, as a function of ${\dot{\gamma }}$, the fraction of all surface-localized beads that roll, *f*_roll_, to quantify whether the bonds in the interface weaken (slip) or strengthen (catch) under mechanical loading. We started with a control experiment consisting of a simple 7-nt slip-bonding DNA duplex. For this experiment, *f*_roll_ increases with increasing shear rate (Fig. 2c and Supplementary Video 3). This is as expected—the increased tension weakens the slip bonds and thus enhances the supramolecular reaction kinetics and thereby promotes rolling.

Strikingly, beads functionalized with our catch bonds show the opposite (Fig. 2c and Supplementary Video 4)—the larger the applied tension, the fewer particles roll and the more particles become stuck to the chip. This is a clear demonstration that our DNA catch-bond design directly strengthens the interface under increasing shear forces. This increase in interfacial stability under increasing shear stress is only possible if the bonds themselves gain mechanical strength under increasing tension. Hence, we can conclude that our DNA construct is the first realization of an artificial catch bond.

**Extended Data Fig. 6:**
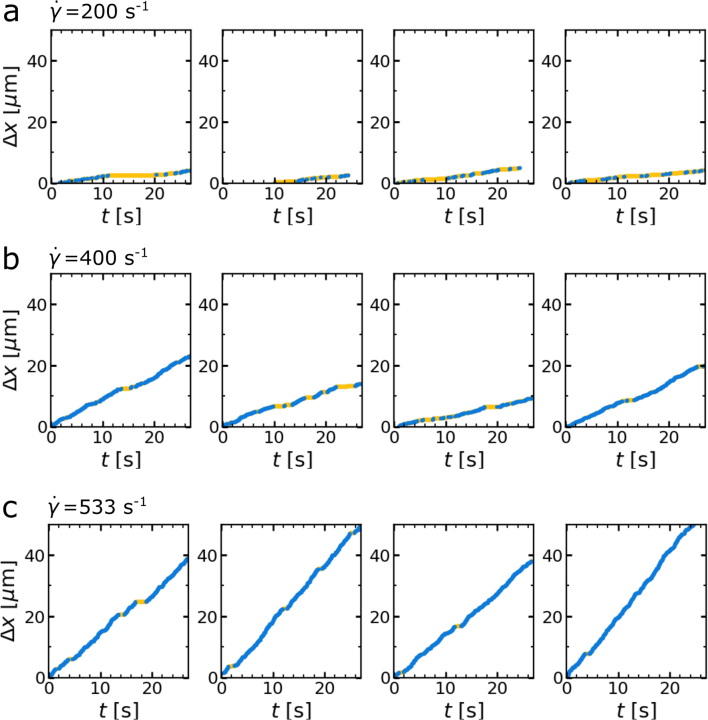
Selection of experimental displacement traces of slip bonded particles. Overview of a few typical displacement traces recorded for slip bonded particles at shear rates *γ*^⋅^ of (a) 200 s^−1^, (b) 400 s^−1^, and (c) 533 s^−1^. Rolling regimes are shown in blue, and stopped regimes in yellow.

**Extended Data Fig. 7:**
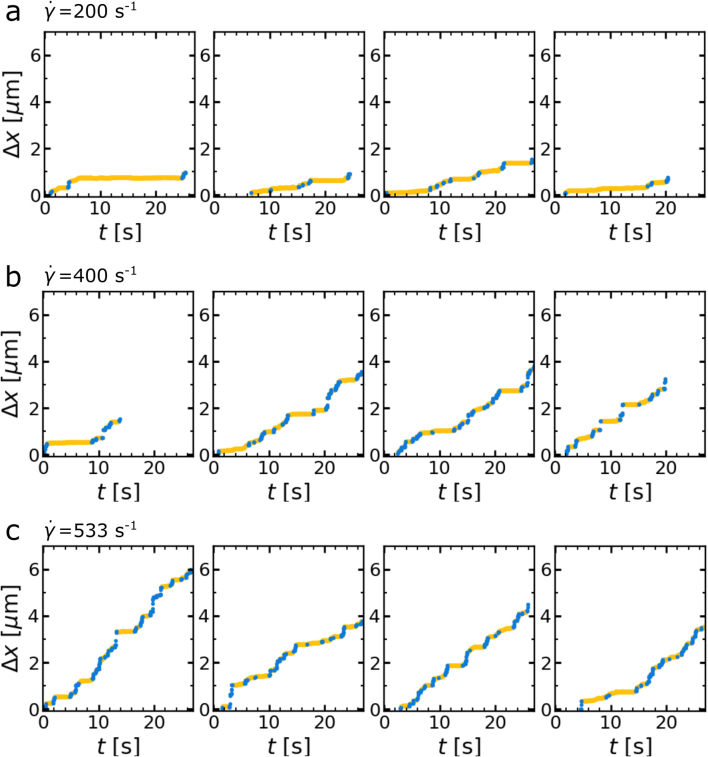
Selection of experimental displacement traces of catch bonded particles. Overview of a few typical displacement traces recorded for catch bonded particles at shear rates *γ*^⋅^ of (a) 200 s^−1^, (b) 400 s^−1^, and (c) 533 s^−1^. Rolling regimes are shown in blue, and stopped regimes in yellow.

The catch-bond nature of our de novo design is manifested also in other features of the same experiments. The displacement trajectories of the rolling particles show distinct intermittency, with alternating periods of rolling and arrest (Fig. 2d). This behaviour is characteristic of rolling adhesion by supramolecular bonds and is the result of stochastic fluctuations in the contact valency^38,65^. We observe this intermittency for both slip and catch bonds, but with distinct differences. For slip bonds, we observe a steep acceleration in motion with increasing ${\dot{\gamma }}$ (Extended Data Fig. 6). By contrast, the rate of motion for catch-bonded particles appears almost insensitive to changes in ${\dot{\gamma }}$ (Extended Data Fig. 7). To quantify these effects, we segmented the trajectories into rolling and stopping regimes to extract their rolling velocity, *v*_roll_, and stop times, *t*_stop_.

**Extended Data Fig. 8:**
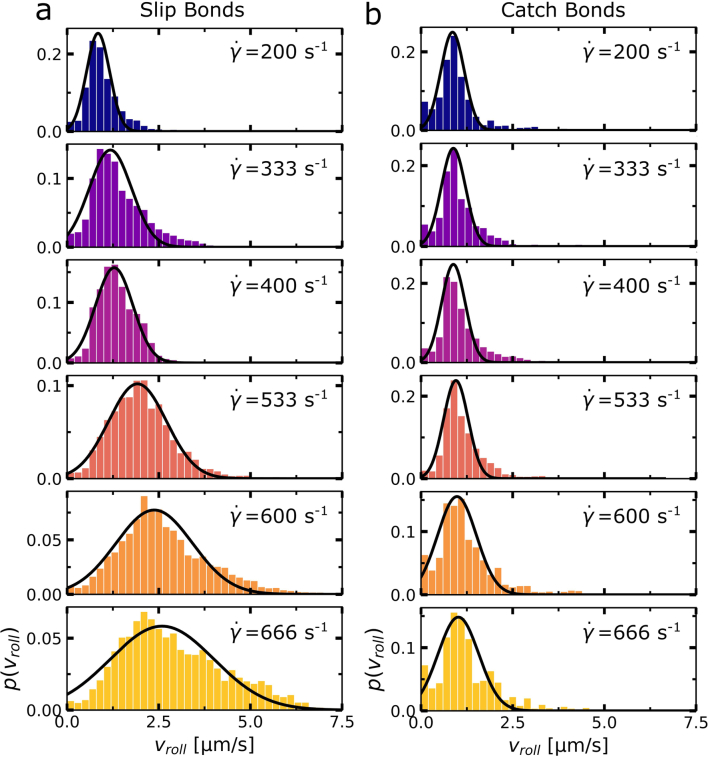
*v*_*roll*_ histograms for particles bound by (a) slipbonds and (b) catch bonds under increasing *γ*^⋅^. Histograms are generally well-described by a Gaussian distribution (black lines). Distributions for slip bonded particles broaden and move to higher *v*_*roll*_ at increasing *γ*^⋅^, while distributions of catch bonded particles remain stable at lower values of *v*_*roll*_.

The rolling velocity of slip-bonded particles increases strongly with shear rate. This is as expected—tension accelerates bond rupture and thereby increases the rate by which the bead can roll along the interface. Interestingly, the rolling velocity of the catch bonds is insensitive to changes in tension (Fig. 2e and Extended Data Fig. 8). This is another manifestation of catch bonding, where the interconversion of catch bonds to the strong state under increased force, which strengthens the interface, compensates the acceleration of the particles under increased shear rates, resulting in a velocity that is insensitive to shear. Besides the rolling velocity in the rolling regime, we also determined the overall average particle velocities, taking into account both the rolling and stopping regimes, and found a similar insensitivity to shear for catch-bonded particles (Supplementary Fig. 5). Catch bonds here thus allow a decoupling of the shear rate and the rate of particle motion. Also the average time particles remain stationary; the stopping time, *t*_stop_, is greater for catch bonds than for slip bonds (Fig. 2f). We conjecture that the stochastic interconversion of a small number of catch bonds to the strong state 2 may result in a temporarily stabilized interface that results in longer pauses.

We note that the distributions in *v*_roll_ and *t*_stop_ are broad, which can be caused by both stochastic fluctuations intrinsic to the system and experimental uncertainty. To assess the extent of the intrinsic stochastic fluctuations, we compared the *v*_roll_ distributions shown per rolling segment to those shown per particle trajectory, in which the intrinsic fluctuations are averaged out (Supplementary Fig. 6). We found that averaging the per particle trajectory removes many of the high and low *v*_roll_ outliers, confirming that much of the experimental uncertainty is caused by intrinsic stochastic fluctuations. We also performed a series of control experiments to identify the causes of the remaining experimental uncertainty and found that variations in bond density on both the channel and particle surfaces account for many of the fluctuations we observe (Supplementary Section Sources of experimental fluctuations).

**Fig. 3:**
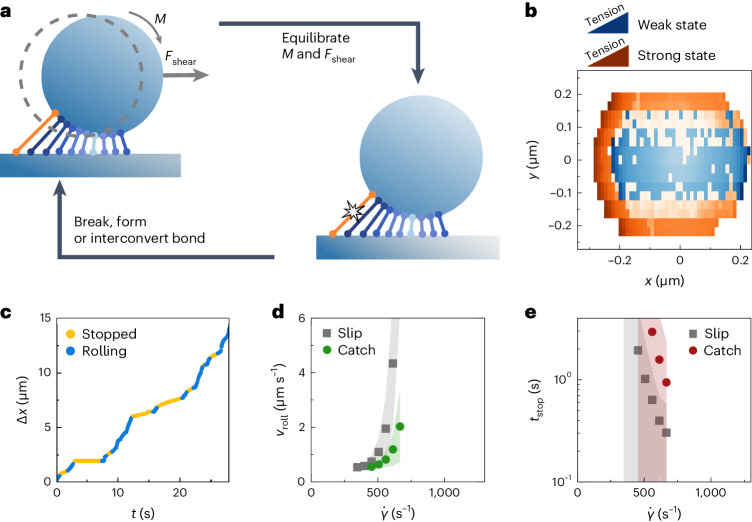
Rolling adhesion simulations highlight the mechanism of interface reinforcement by catch bonds under shear. **a**, Schematic of the simulation protocol followed by one kinetic Monte Carlo step in which a bond activates, forms or dissociates. By repeating these steps, we simulate a rolling particle over time. **b**, Top–down heatmap at ${\dot{\gamma }}={560}\,{{{{\rm{s}}}}}^{-1}$, showing the spatial distribution of catch bonds in the weak and strong states relative to the centre of the particle. The colour intensity indicates the average extensional force on the bonds. Catch bonds primarily activate in a small region at the trailing end of the particle, where tension is highest. **c**, A simulated displacement trace of a catch-bonded particle shows intermittent stop–roll behaviour. **d**, A plot of the average rolling velocities, *v*_roll_, as a function of ${\dot{\gamma }}$ reveals that catch bonds impart a lower sensitivity to the shear rate compared to slip bonds. **e**, Particle stopping times are higher, on average, for catch-bonded particles over the range of ${\dot{\gamma }}$ evaluated. Shaded zones in **d** and **e** indicate standard deviations.

The results from our rolling adhesion experiments suggest that catch bonds can enhance the stability of the rolling adhesive interface by decreasing the sensitivity of the particle–surface interface to the shear rate. For a more mechanistic insight into this effect, we established a model for rolling adhesion based on steady-state kinetic Monte Carlo simulations (Fig. 3a). These simulations allow us to predict the particle displacement trajectories, taking as input a molecular mechanotype, such as a slip-bond versus catch-bond mechanochemical interaction. Catch bonds in the interface are simulated as being in either the weak or strong state. The shear field at the surface results in both a shear force (*F*_shear_) and torque (*M*) acting on the particles^36^. This displaces the particle, thereby stretching the bonds until mechanical equilibrium is reached. A stochastic algorithm then breaks, forms and interconverts the individual bonds in the microparticle–surface interface, based on a force-dependent Kramers–Bell-type model for either faster (weak state and slip bond) or slower (strong state) dissociation under tension. A detailed description of the simulation procedure and the assumptions made is provided in Supplementary Section Rolling adhesion simulation protocol. By simulating a rolling microparticle in this way, we can reproduce many of the features in the experiments, but with additional insight into what happens at the molecular level.

A heatmap that shows the distribution of catch bonds in the weak versus activated states (${\dot{\gamma }}={560}\,{{{{\rm{s}}}}}^{-1}$) reveals a clear localization of activated (strong) catch bonds along a small ridge at the trailing edge of the particle, where tension on the interface stress is highest (Fig. 3b). This strong localization of catch-bond activation in zones of high stress is also thought to contribute to their effectivity in promoting cohesive stability in networks^9,29^.

**Extended Data Fig. 9:**
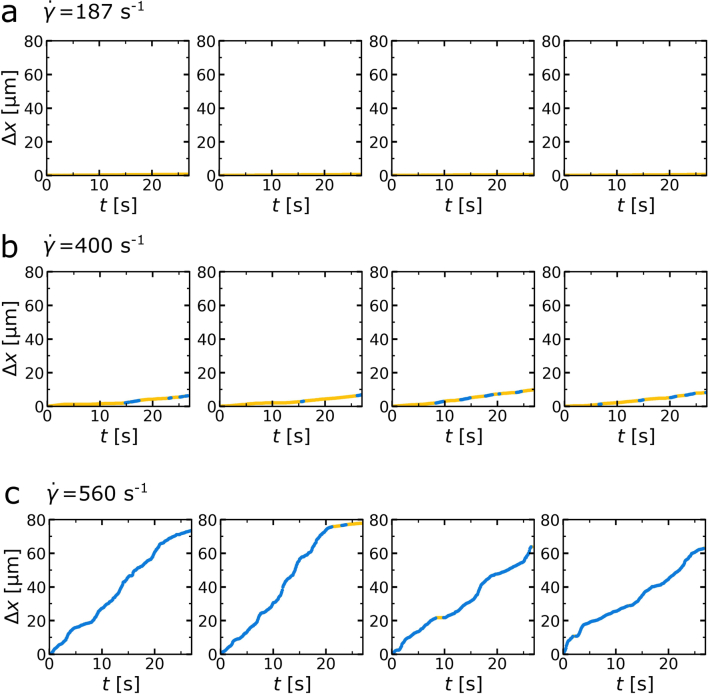
Selection of simulated displacement traces of slip bonded particles at shear rates *γ*^⋅^ of (a) 187 s^−1^, (b) 400 s^−1^, and (c) 560 s^−1^. Rolling regimes are shown in blue, and stopped regimes in yellow.

**Extended Data Fig. 10:**
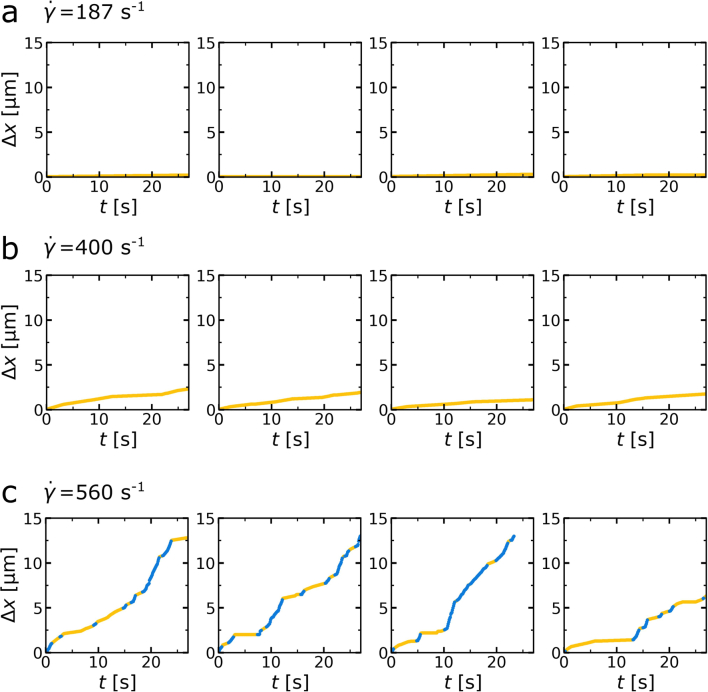
Selection of simulated displacement traces of catch bonded particles at shear rates *γ*^⋅^ of (a) 187 s^−1^ (b) 400 s^−1^, and (c) 560 s^−1^. Rolling regimes are shown in blue, and stopped regimes in yellow.

We performed simulations with both slip and catch bonds, and in both cases found intermittent stop–roll behaviour, similar to that observed in the experiments (Fig. 3c and Extended Data Figs. 9 and 10). Similar to the experimental data analysis, we obtained the rolling velocity, *v*_roll_, and stopping time, *t*_stop_, as a function of shear rate (Fig. 3d,e). We found that these trends reproduce the experimentally observed *v*_roll_ and *t*_stop_ data (Fig. 2d,e), using experimentally obtained mechanochemical parameters for the slip- and catch-bond DNA interactions as input parameters. This further verifies that all of the features described above in the experiment are direct manifestations of catch bonding in our DNA designs. A complete list of the simulation parameters and their justification is provided in Supplementary Section Rolling adhesion simulation protocol. At large shear rates, the simulated *v*_roll_ increases exponentially for both the slip-bond and catch-bond data (Fig. 3d). Although we do not observe this exponential increase in our experimental *v*_roll_ data (Fig. 2d), we expect this should take place at larger shear rates, which are inaccessible due to the practical limits in our flow control. The *v*_roll_ histograms of the slip-bond simulations show similar broadening at increasing shear rates compared to the experimental data. Simulated *v*_roll_ distributions are furthermore narrower than the corresponding experimental distributions (Supplementary Fig. 7). We believe, here, that the terminal regime of the catch bond is reached, where the strong state of the catch bond is overpowered by force, causing the catch bond to revert back to slip-bond behaviour.

Previous rolling adhesion studies on protein catch bonds have identified a regime in which the rolling velocity decreases with increasing shear rate^36^. To determine whether this regime is accessible in our simulations, we performed an additional simulation series in which we systematically explored the parameter space in terms of the bond dissociation rates from the weak and strong states, and the mechanical susceptibility towards catch-bond activation (Supplementary Fig. 8). Despite substantially varying these parameters and explicitly including catch-bond behaviour, we do not observe a regime of decreasing rolling velocity. We therefore conclude that the decrease in *v*_roll_ is not a feature of our synthetic DNA catch bond within the regimes we can access.

**Fig. 4:**
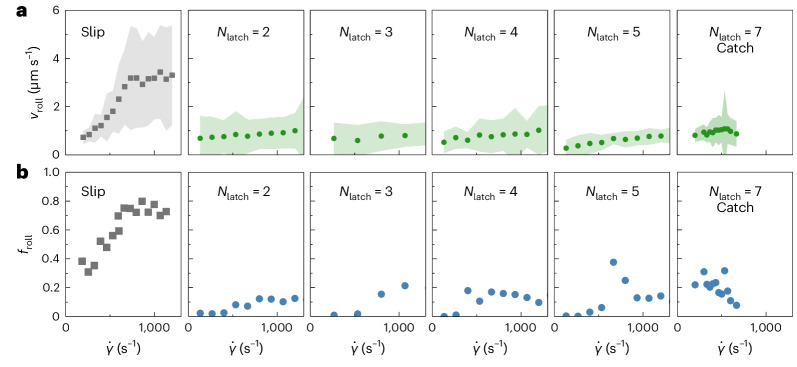
Variations in latch sequence length reveal the emergence of catch-bond behaviour at high *N*_latch_. **a**, Plots of the average *v*_roll_ as a function of ${\dot{\gamma }}$ over a range of *N*_Latch_ sequence lengths (green) in comparison to the slip bond (grey). Contrary to the slip-bond control, *v*_roll_ is insensitive to ${\dot{\gamma }}$ for each latch sequence variation. This indicates a deviation from slip-bond behaviour similar to that observed for the catch-bond design for *N*_latch_ = 7. **b**, Plots of *f*_roll_ as a function of ${\dot{\gamma }}$ over a range of *N*_latch_ sequence lengths (blue) and the slip bond (grey). For *N*_latch_ between 2 and 5, *f*_roll_ increases slightly or remains constant. By contrast, a clear decreasing trend indicates catch bonding is only observed for *N*_latch_ = 7. Filled zones in **a** indicate standard deviations. Measurements for *N*_latch_ = 7 at ${\dot{\gamma }} > {667}\,{{{{\rm{s}}}}}^{-1}$ contained fewer than 30 particles and were hence excluded due to a lack of statistics. The numbers of particle trajectories analysed per datapoint are listed in Supplementary Tables 5b and 6a.

Theoretical predictions and experimental evidence on biological catch bonds^15,66^ suggest that, between slip bonds and catch bonds, a regime exists where ‘ideal’ bonds can be found. Ideal bonds neither weaken nor strengthen under tension, but their stability is insensitive to the applied force. In our catch-bond design, the 7-nt latch sequence strengthens the bond under tension. To explore the regime between slip and catch bonds in our design, we systematically varied the length of the latch sequence *N*_latch_, expecting to gradually weaken the strong state. We found that latch lengths of 2–5 nt result in a rolling velocity that is invariant to the applied shear stress, whereas for slip bonds it increases substantially (Fig. 4a). Clearly, all designs with a latch of 2 nt or more do not behave as conventional slip bonds. However, these designs did not show the hallmark feature of catch bonds, which is a decreasing fraction of mobile particles, *f*_roll_, with increasing shear rate. Rather, within the experimental noise, *f*_roll_ appears constant or increases weakly with imposed hydrodynamic force. This behaviour, in between catch and slip bonds, suggests ideal bonding. This illustrates how, in a single design, we can gradually tailor the molecular mechanics from slip to catch bonds, through an intermediate regime that is reminiscent of ideal bonding. At the moment, we do not fully understand the exact origin of the role of this latch length in the switch in slip–ideal–catch bonding behaviour. Nevertheless, it illustrates the narrow phase space in which true catch bonding is observed, which can be disrupted with a minimal design change.

## Conclusion

We have reported on the design and realization of an artificial de novo catch bond built using DNA duplexes. Despite several theoretical efforts to facilitate rational design in the past^32,33^, an experimental realization had not yet been achieved. Starting from the conceptual framework of the two-state, two-pathway catch bonds found in nature^19,41,42^, and its abstraction into four tangible design requirements, we have proposed a DNA-based construct capable of tension-induced and reversible switching between a thermodynamically stabilized weak state and a mechanically stabilized strong state. Moreover, we have shown how collectives of these catch bonds, in direct analogy to their natural counterparts during leukocyte rolling^36,37^, can promote the adhesive strength of an interface under tension, whereas slip bonds weaken as they experience increasing forces. This Article introduces catch bonds into the synthetic domain, opening up a plethora of opportunities to create DNA-origami-based nanomachines and other man-made catch-bonded materials. These are theoretically predicted to result in fascinating features, including the unification of structural plasticity with mechanical resilience^9^, improved fracture resistance and toughness^29,31^ and mechano-adaptivity^67^.

## Methods

All chemicals were purchased from Sigma Aldrich, unless stated otherwise.

### DNA duplexes

DNA oligonucleotides were purchased from Integrated DNA Technologies, and their sequences are reported in Supplementary Table 1. In the rolling adhesion experiments, we utilized catch-bond (CB-HS and CB-CS) and slip-bond (SB-1 and SB-2) sequences that each contained a 5′ biotin group for attachment to streptavidin, followed by a 40-nt spacer sequence to repress non-specific interactions and finally the target bond sequence. A filler sequence (filler) containing a 5′ biotin, the 40-nt spacer and a T5 end was mixed together with the catch- or slip-bond sequences to tune the density of available bonds on the microfluidic channel surface. In the oxDNA simulations, we simulated only the target bond sequence, without the 40-nt spacer sequence, for computational efficiency.

The following buffer solutions were used during sample preparation:

- A: 10 mM Tris, 1 mM EDTA, 2 mM MgCl_2_, pH 6
- B: buffer A, containing 0.1 mg ml^−1^ BSA, 0.2% wt/vol Pluronic F108, pH 6
- C: buffer B, containing 100 mM NaCl, 0.03 mg ml^−1^ biotin, pH 6

First, DNA oligos (CB-HS, CB-CS, SB-1 or SB-2) were hybridized to the spacer-complement sequence by combining 10 μM target and 10 μM spacer-complement in buffer A. The mixtures were heated for 5 min at 95 °C and gradually cooled to room temperature over 1.5 h. Hybridized oligos were kept at 4 °C and used within 24 h for functionalization.

### Surface functionalization

All surface functionalization steps and rolling adhesion experiments were performed at 20 °C. Streptavidin-coated polystyrene microparticles (1.36-μm diameter, Spherotech Inc.) were first transferred to buffer B as follows: 50 μl of a 0.5% wt/vol particle suspension was centrifuged for 6 min at 12,000*g*, the supernatant was removed with a pipette and the particles were resuspended in 1 ml of buffer B. This washing–resuspension cycle was repeated three times. During the final cycle, particles were resuspended in 80 μl of hybridized slip- or catch-bond oligos (CB-CS or SB-2, or latch variations CB-CS-L2 to CB-CS-L5; Supplementary Table 1) and incubated at room temperature for 5 min. Finally, functionalized particles were washed by three more centrifugation–resuspension cycles using 1 ml of buffer B. On the final cycle, particles were resuspended in 500 μl of buffer C, which contained free biotin to irreversibly block any remaining unoccupied streptavidin sites and prevent non-specific interactions arising from these. Finally, the particle suspension was diluted to the final concentration by adding 30 μl of the particle suspension to 470 μl of buffer C. This suspension was stored at 4 °C until further use within two days.

Rectangular microfluidic channels (1,500 μm × 50 μm × 40 mm, Micronit Microtechnologies BV, cat. no. FC_FLC50.3) were functionalized with the hybridized target DNA (CB-HS or SB-1) using a sequential functionalization with biotinylated BSA, streptavidin and finally DNA, as follows. First, the channels were mounted in a chip holder (Micronit Fluidic Connect Pro) and connected with polypropylene tubing (1/32-inch inner diameter; 1/16-inch outer diameter). Before functionalization, the chip was filled with 1 M KOH for 5 min to etch the channel surface and thoroughly flushed with 4 ml of Milli-Q water until neutralized. The chip holder was then mounted onto a Nikon Ti2 Eclipse brightfield microscope and connected to an Elveflow Sequential Fluid injection set-up (Elvesys) composed of a pressure controller (OB1 Mk3+), a flow rate sensor (MFS3-80) and a 12-way distribution valve to sequentially inject different solutions (MUX). All of the following functionalization steps were performed with the chip mounted in this set-up, immediately before the rolling adhesion experiment. First, 0.5 ml of a biotinylated BSA (Pierce) solution (1 mg ml^−1^) in buffer A was introduced into the channel, followed by a 5-min incubation at a flow rate of 0 μl min^−1^. Buffer A is slightly acidic (pH 6) to facilitate better absorption of the BSA to the glass surface^68^. Next, the channel was flushed with 5 ml of buffer B to remove any remaining unbound biotinylated BSA. The channel was then functionalized with streptavidin (ThermoFisher) by flowing 0.5 ml of a streptavidin solution (0.02 mg ml^−1^) in buffer A through the channel at 50 μl min^−1^. After another flushing step with 5 ml of buffer B, a mixture of 0.15 ml of 10 μM hybridized DNA (CB-HS or SB-1) and 0.15 ml of 10 μM hybridized filler DNA and 0.6 ml buffer B was introduced to the channel, followed by 5 min of incubation at 0 μl min^−1^. This 1:1 ratio of target DNA and filler DNA was found to be optimal for achieving stable rolling behaviour. Finally, the remaining excess of DNA was removed by flowing 5 ml of buffer B.

### Rolling adhesion assays

After functionalizing the surface of the microfluidic chips with the hairpin strand of the catch bond, and attaching the complementary strand to the microparticles, we performed our rolling adhesion assay as follows. Within 1 h after microfluidic channel functionalization, a batch of functionalized microparticles was introduced into the functionalized microfluidic channel. The flow was switched to 0 μl min^−1^ for 5 min to allow the particles to settle to the bottom of the flow cell. Sedimented particles in the centre of the channel were brought into focus under a brightfield microscope, using a ×40 objective (Nikon Plan apo lambda) and a Fastec HiSpec 2G Mono camera. We then used the Elveflow microfluidic flow controller to apply well-defined shear rates to the particles. To begin a measurement, the microfluidic flow rate was set to the flow rate of interest and allowed to stabilize, after which a movie of 1,634 frames was recorded at 60 f.p.s., using a resolution of 1,280 × 1,024 pixels. In a typical measurement, we imaged between 100 and 400 particles in the field of view at a time. Measurements were repeated for a range of flow rates, *Q*. The imposed flow rate was converted to the wall shear rate ${\dot{\gamma }}$ assuming Poisseuille flow:

1 $${\dot{\gamma }}={\frac{6Q}{{h}^{2}w}}$$

where *h* denotes the height of the channel and *w* the width of the channel. Equation (1) is a valid approximation for channels containing a Newtonian fluid with rectangular shape and *w* ≫ *h*. The recorded movies were analysed using a custom-built Python particle-tracking script that employs the TrackPy package^69^, which has been made publicly available at https://github.com/jorissprakel/DNACatchBond. Before particle tracking, the raw movies were pre-processed to convert the brightfield spots into trackable Gaussian features through a series of steps. First, an intensity threshold step was used, setting all values below a threshold intensity to 0. This threshold step removed the out-of-focus particles flowing past and the dark rings surrounding each particle spot, leaving only the bright spots at the centres of the particles. This resulted in bright, truncated features. The threshold value was determined manually for each dataset to ensure that it was higher than the background intensity of out-of-focus particles, but lower than the maximum intensity of the in-focus particles. Threshold values of between 25 and 45 were used. Next, a Gaussian blur (width 1 pixel) was used to transform the truncated features into Gaussian spots, which were then tracked by the TrackPy locate function. Particles that displaced more than 1 μm over the course of their trajectories were considered rolling and included in the fraction of rolling particles *f*_roll_. Only those particles tracked for at least 30 frames were considered in the analysis, and datasets containing fewer than 30 traced particles were excluded from the analysis owing to a lack of statistics. Trajectories of the rolling particles were separated into stopping and rolling regimes using an instantaneous velocity threshold of 0.5 μm s^−1^, which was empirically found to provide good separation between the regimes. A Savitzky–Golay filter was used to smooth noise in the instantaneous velocity profile and allow for a more precise determination of the regimes. Finally, the average stop time, *t*_stop_, was computed from the stopping regime duration. The average rolling velocity, *v*_roll_, was computed per particle trajectory by computing the average velocity over all rolling regimes experienced by the particle.

### Rolling adhesion simulations

Rolling adhesion simulations were performed with a custom-developed Python program. The simulation and data-analysis code has been made publicly available at https://github.com/jorissprakel/DNACatchBond. A thorough description of the simulation protocol and motivation for the parameters is provided in Supplementary Section Rolling adhesion simulation protocol. Our simulations made use of a Gillespie kinetic Monte Carlo algorithm to determine which step took place at a given moment (bond dissociation, bond formation or bond activation). Single-particle simulations were performed on particles bound via slip bonds and particles bound via catch bonds, over a range in shear rates ${\dot{\gamma }}$. Each simulation was run for a total of 5 × 10^5^ simulation steps and was repeated 50 times. As the Kinetic Monte Carlo simulations are event-driven, the displacement data are inhomogeneously distributed in time. For this reason, displacement data were linearly interpolated over time, using an interpolation time step of 0.01. The interpolated displacement traces were split into rolling and stopped regimes using an instantaneous velocity cutoff of 0.5, which was empirically found to provide good separation between the regimes. A Savitzky–Golay filter was again used to smooth noise in the instantaneous velocity and allow for a more precise determination of the regimes. From these separated regimes, the average stopping times of the stopping regimes were determined. Finally, the average rolling velocity was computed from the rolling regimes, weighted following the duration of the regimes. To generate the catch-bond activation heatmap at high shear, a separate set of 100 simulations were performed for a total of 5 × 10^5^ simulation steps at ${\dot{\gamma }}={560}\,{{{{\rm{s}}}}}^{-1}$, during which the position, state and extensional force on each linker were stored every 2,500 steps. The catch-bond activation heatmap (Fig. 3b) was then generated as follows. First, a two-dimensional array of bins was defined on the channel surface, relative to the centre of the particle. Within each bin, the average extensional force on catch bonds in the weak and strong states was determined over the course of the simulation. Averaging the heatmaps further over the 100 simulated repeats resulted in the activation heatmap in Fig. 3b.

## Online content

Any methods, additional references, Nature Portfolio reporting summaries, source data, extended data, supplementary information, acknowledgements, peer review information; details of author contributions and competing interests; and statements of data and code availability are available at 10.1038/s41557-024-01571-4.

## Supplementary information

Supplementary Information DNA sequence used in the study, Discussion, Rolling adhesion simulation protocol, videos, Rolling adhesion particle statistics, Tables 1–6 and Figs. 1–20.

Supplementary Video 1 Render of an oxDNA MD simulation showing a catch bond interconverting from state 1 to state 2 under 30 pN of overall tension. Under tension, the hairpin opens quickly, which is then followed by a closing of the latch to yield state 2. The simulation consisted of 1.6 × 10^9^ MD steps in total, and a snapshot is shown every 2 × 10^6^ simulation steps.

Supplementary Video 2 Render of an oxDNA MD simulation showing a catch bond relaxing from state 2 to state 1 in the absence of tension. Initially, the toe hold forms rapidly, followed by an internal strand exchange that displaces the complementary strand and reforms the hairpin to yield state 1. The simulation consisted of 1.6 × 10^9^ MD steps in total, and a snapshot is shown every 2 × 10^6^ simulation steps.

Supplementary Video 3 Close-up of a typical rolling adhesion experiment using slip bonds (SB-1 and SB-2) performed at *γ*^⋅^ = 400 s^−1^ (left) and *γ*^⋅^ = 667 s^−1^ (right). Particle traces are shown as yellow lines, and tracked particles are colour-coded based on whether they are in a rolling regime (green) or stopping regime (red). A clear increase in rolling velocity is observed at increasing *γ*^⋅^.

Supplementary Video 4 Close-up of a typical rolling adhesion experiment using catch bonds (CB-HS and CB-CS) performed at *γ*^⋅^ = 400 s^−1^ (left) and *γ*^⋅^ = 667 s^−1^ (right). Particle traces are shown as yellow lines, and tracked particles are colour-coded based on whether they are in a rolling regime (green) or stopping regime (red). At *γ*^⋅^ = 400 s^−1^, a larger fraction of particles display rolling behaviour than at the higher shear rate of *γ*^⋅^ = 667 s^−1^.

## Data Availability

The raw data associated with the figures in this manuscript are publicly available at 10.4121/96e43d14-80a6-46e2-819c-9c627cedf10e.v2.

## Code availability

All simulation and data analysis codes used to perform and analyse the work in this article have been made publicly available at https://github.com/jorissprakel/DNACatchBond/.
