# Supplementary material for: De novo DNA-based catch bonds
Source: Nat Chem. 2024 Jun 24;16(12):1943–50. doi: 10.1038/s41557-024-01571-4 (PMC11611730; doi:10.1038/s41557-024-01571-4)
Supplement: Supplementary file 1 — DNA sequence used in the study, Discussion, Rolling adhesion simulation protocol, videos, Rolling adhesion particle statistics, Tables 1–6 and Figs. 1–20. [file 41557_2024_1571_MOESM1_ESM.pdf]

nature chemistry

Supplementary information

https://doi.org/10.1038/s41557-024-01571-4

De novo DNA-based catch bonds

In the format provided by the authors and unedited

Contents

Overview of DNA sequences used in this study 2

FRET experiments on the *de novo* catch bond 3

Coarse-grained molecular dynamics simulations 5

Sources of experimental fluctuations 6

Rolling adhesion simulation protocol 10

List of supplementary videos 14

Particle numbers 15

References 19

Supplementary Figures 20

Table S1. DNA sequences used in this study, in 5' to 3' orientation. The domains in our catch bond design are labelled as follows: Barrier sequence (green), latch (yellow), toe-hold (purple), and cross-link (gray).

Label	Sequence
Spacer (Filler)	Biotin-TTTT-GCGTATGTCCTCGGATGGTTCAGTTGTAACGA TATACTTA-TTTT
Spacer-C	TAAGTATATCGTTACAACTGAACCATCCGAGGACATACGC
**Slip bond**	
SB-1	Spacer–TGTACGCC
SB-2	Spacer–GGCGTACA
**Catch bond**	
CB-HS	Spacer-CGGCGCATGATAGATTCAGTTTTCTGAATCTATCATG CGCCGCACGAAGAGA
CB-CS	Spacer-TCTCTTCGTTTTTTTTATGATAG
**Latch variations**	
CB-CS-L2	Spacer–TCTCTTCGTTTTTTTTAT
CB-CS-L3	Spacer–TCTCTTCGTTTTTTTTATG
CB-CS-L4	Spacer–TCTCTTCGTTTTTTTTATGA
CB-CS-L5	Spacer–TCTCTTCGTTTTTTTTATGAT
**Labelled spacers**	
A488-Spacer	Alexa488-TTTT-TAAGTATATCGTTACAACTGAACCATCCGAG GACATACGC
A565-Spacer	Atto565 -TTTT-TAAGTATATCGTTACAACTGAACCATCCGAG GACATACGC
**Barrier variations**	
CB-HS-var1	Spacer-CGGCGCATGATAGATTCAGTTTTTACTGAATCTATC ATGCGCCGCACGTTGACG
CB-CS-B0	Spacer-CGTCAACGTCCGGCG
CB-CS-B1	Spacer-CGTCAACGTTTGGCGC
CB-CS-B2	Spacer-CGTCAACGTTTTGCGCA
CB-CS-B3	Spacer-CGTCAACGTTTTTCGCAT
CB-CS-B4	Spacer-CGTCAACGTTTTTTGCATG
CB-CS-B5	Spacer-CGTCAACGT TTTTTTCATGA
CB-CS-B6	Spacer-CGTCAACGT TTTTTTTATGAT

FRET experiments on the *de novo* catch bond

We performed Förster Resonance Energy Transfer (FRET) experiments to confirm that the catch bond construct assembles in the weak state 1 when no tension is applied. To this end, we designed and ordered Alexa Fluor-labelled DNA oligonucleotides that are complementary to the 40 nt spacer sequence present on the 5' ends of the hairpin strand and complementary strand of the catch bonds (CB-HS and CB-CS). The DNA sequences of these oligos are listed in table S1. When hybridized to the DNA catch bond construct, the two Alexa Fluor dyes are in close proximity and hence show FRET activity (Extended Data Fig. 1a). As the distance between the dyes is much larger in the state 2 (strong) compared to the weak state 1 (weak), we can use the FRET efficiency E as a ruler to determine the state of the catch bond. FRET experiments were performed on five catch bond designs with varying latch sequence lengths N_{Latch} ranging from 2 to 7 nt. To vary the latch sequence, we changed the length of the latch on the complementary strand (sequences CB-CS-L2 to L5, table S1), while using the same hairpin strand (CB-HS). $N_{Latch} = 7$ corresponds to our main catch bond design (CB-HS and CB-CS).

A FRET experiment was performed as follows: Fluorescently labeled probes were hybridized separately to the CB-HS and CB-CS sequences at 2 µM using the DNA hybridisation protocol described in the Materials and Methods section. After hybridization, the two hybridized DNA complexes were mixed in a 1:1 ratio. We imaged 10 µl of sample under a Leica SP-8 inverted confocal microscope, using an SPC830 time-correlated single-photon counting module (Becker & Hickl) for 120 seconds to acquire the fluorescence lifetime histogram. Average fluorescence lifetimes were obtained by parabolic fitting of the top half of the peaks in these histograms with a parabolic function. Each FRET measurement was performed in duplo, and the obtained FRET efficiencies are shown in Extended Data Fig. 1b. We determined whether these FRET efficiencies correspond to the weak or strong state of the catch bond through coarse-grained molecular dynamics simulations using the oxDNA package (See also SI section *Coarse-Grained molecular dynamics simulations*). For each of the catch bond sequences, we performed $1 \cdot 10^6$ simulation steps, after which an expected average FRET efficiency was computed using the TacoxDNA package [1], taking into account both the configuration of the DNA strands during the simulated trajectory, and the orientational freedom of the fluorophores. For each of the catch bond sequences, we performed $1 \cdot 10^6$ simulation steps and acquired a trajectory of $1 \cdot 10^3$ configurations. These configurations were converted into a PDB format using TacoxDNA [1]. The PDB files were used as input for the python package LabelLib to simulate the fluorophores on the DNA conformation [2]. The fluorophores were attached to the O-5' atom on the 5' end of the spacer sequence. Similar to the experimental conditions, we hybridized the A488-Spacer sequence to the hairpin strand and the A565-spacer sequence to the complementary strand. The dimensions of the fluorophores were estimated using Chemdraw 3D. The linker length was estimated to be 13.5 by 2.5 Angstroms, the AlexaFluor molecule size was 4 by 4.5 by 1.5 Angstroms,

and the ATTO 565 molecule size was 9 by 7 by 4.5 Angstroms. For each conformation $1 \cdot 10^6$ randomly sampled distances between the fluorophores were averaged. The average distance was converted into FRET efficiency E using: $E = \frac{1}{\left(1+(r/r_F)^6\right)}$, with $r_F = 6.4$ nm the Förster radius of the Alexa488-Atto565 dye pair.

Strikingly, we find that the observed experimental FRET efficiencies are very close to the simulated predictions for the catch bond in the inactivated state, for all of the N_{Latch} values we evaluated (Extended Data Fig. 1b). This provides strong evidence that our construct forms the weak state 1 upon assembly, and hence that weak state 1 is the preferred state in absence of tension. We performed a further series of FRET measurements in which we varied the length of the barrier sequence (Extended Data Fig. 1c). We performed a series of FRET measurements on a variation of the CB-HS strand (CB-HS-var1, Table S1), and a series of variations on the complementary strand, containing a variable number of T-nucleotides between the latch sequence and the cross-link sequence that acts as the barrier (sequences CB-CS_B0 to CB-CS_B6). In this way, we varied the number of nucleotides in the barrier sequence $N_{Barrier}$ from 0 to 6. We find that the catch bond spontaneously assembles in the strong state if the barrier sequence is absent ($N_{Barrier} = 0$ nt), while it spontaneously assembles in the weak state at $N_{Barrier} \geq 5$ nt. A barrier sequence of at least 5 nt was therefore found essential to shift the thermodynamic equilibrium of the catch bond towards the weak state and hence fulfil design requirement IV of a synthetic two-state catch bond.

Coarse-grained molecular dynamics simulations

We tested the force-induced mechanism of action of our *de novo* catch bond using coarse-grained molecular dynamics simulations with the oxDNA model [3, 4]. The oxDNA model has previously been found to allow for efficient simulation of DNA bases, while simultaneously offering an accurate description of the thermodynamic and mechanical properties of DNA base-pairing interactions. This makes it an excellent model to evaluate how our DNA catch bond responds to mechanical load.

We first simulated the activation mechanism of the catch bond under tension. Starting from an equilibrated conformation in the weak state 1 of the catch bond, we stretch the catch bond by applying a constant external stretching force of 30 pN in opposite directions on the final nucleotides of the hairpin strand and complementary strand, using oxDNA's external "string" force setting. A total of 30 activation simulations were run for $1.6 \cdot 10^9$ MD steps using a stepsize of $dt = 0.005$, temperature $T = 20$ C, and salt concentration of 0.1 M. A render of a typical activation simulation is included as supplementary video 1. A trajectory of a typical simulation shows a rapid opening of the hairpin, followed by a closing of the latch to form the strong state 2, see Extended Data Fig. 2a. 14 out of 30 simulations showed this activation within the $1.6 \cdot 10^9$ MD steps.

We next simulated the formation of the weak state 1 in absence of tension, starting from a construct in the strong state 2. A total of 30 relaxation simulations were performed with $8 \cdot 10^8$ MD simulation steps with the OXDNA2 model parameters, using a stepsize of $dt = 0.005$, temperature $T = 20$ C, and salt concentration of 0.1 M were performed. An example of a typical simulation result is shown in supplementary video 2. We find that the construct readily formed state 1 by the end of the simulation. During the simulation, we track the state of the catch bond with the order parameters $f_{toe-hold}$ and f_{latch}, which are the fractions of bound base pairs in the toe-hold region, and bound base pairs between the complementary strand and hairpin strand in the latch region, respectively (Extended Data Fig. 2b). A base-pair is considered bound when it is within 0.5 μm from it's complementary partner. Initially, we observe a quick formation of the toe-hold as evidenced by a jump in $f_{toe-hold}$. Next, f_{latch} fluctuates over time as the hairpin attempts to displace the latch sequence from the complementary strand, until the complementary strand is displaced and the catch bond reverts to the weak state 1. This transition from state 2 to state 1 can further be seen in simulation snapshots (insets in Extended Data Fig. 2b). 17 out of 30 simulations showed this relaxation to state 1 within the $8 \cdot 10^8$ simulation steps.

Sources of experimental fluctuations

Our experimental results contain substantial statistical variation, which can be partially attributed to experimental fluctuations. We have performed a series of control experiments to identify the causes of these experimental fluctuations. First, we investigated bond density variability on both the channel and particle surface as a potential cause, using a control experiment with fluorescently labelled DNA oligos. To this end, we functionalized a batch of 4.34 μm streptavidin-coated polystyrene particles with a DNA duplex containing a sticky end of the sequence: 5'-Spacer-TTTT-ACTGGTCCTGCGGCGTACAT-3', hybridized to the spacer-C sequence. This functionalization was done using the same procedure described in the Materials & Methods section. After functionalization, the particles were incubated for 5 minutes in 80 μL of buffer **B** containing 10 μM of an Alexa488-labelled DNA oligo complementary to the sticky end on the particles, of sequence: 5'-Alexa488-TTTTATGTACGCCGCAGGACCAGT-3'. Next, three washing steps with buffer **C** were performed to remove the excess of the Alexa488 oligo. The functionalized particles were pipetted on a coverslip and imaged using a confocal fluorescence microscopy (Nikon Ti2 Eclipse, 60x objective, excitation: 488 nm, confocor: C2). The channels were functionalized using the procedure described in the Materials & Methods, using a mixture of the sticky-ended DNA oligo described above and the double-stranded spacer, at two ratios: 50% and 100% sticky ended target. After functionalization, we flowed 0.9 mL of a solution of the Alexa488-labelled DNA oligo (3.3 μM) in buffer **C** through the channel, and incubated for 5 minutes by setting a flowrate of 0 μL/min. Next, 4 mL of buffer **C** was flowed through to remove the excess of Alexa488 DNA, and the channels were again imaged using the Nikon confocal microscope with a 4x objective.

Confocal fluorescence images of the fluorescently labelled particles show substantial spatial fluctuations and particle-to-particle fluctuations (Supplementary Fig. 1a). We find similar patchiness in the fluorescence images of the channel surface (Fig. SXX b,c), which is especially clear at 50% sticky-ended target (Fig. SCC b). Considering that particles traverse distances of up to 40 μm over the course of their trajectories, rolling particles will cross several patches of varying bond density on both the channel and particle surfaces. We therefore conclude that fluctuations in bond density are an important source of the fluctuations in the rolling adhesion data. Note that we chose somewhat larger particles in these control experiments as compared to our rolling adhesion experiments (4.34 μm vs 1.36 μm), as these larger particles make observing fluctuations in bond density of the surface more straightforward.

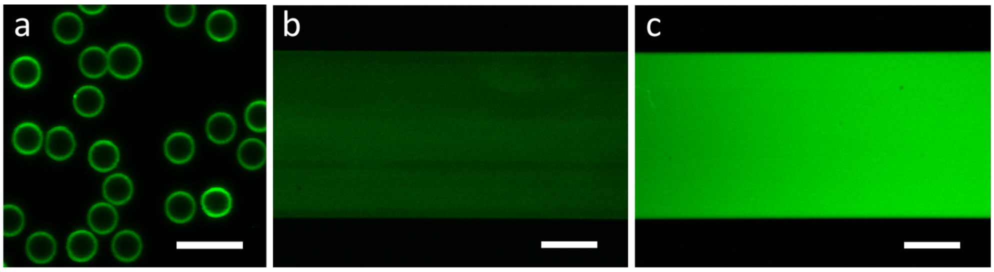

Supplementary Fig. 1. Confocal fluorescence microscopy images, showing (a) particles functionalized with the target DNA followed by hybridization with Alexa488 DNA complement, and (b,c) the microfluidic channel functionalized with 50% and 100% target DNA respectively, followed by hybridization with the Alexa488 DNA complement. The scale bar in a represents 10 μm, and scale bars in b and c indicate 500 μm.

Having observed that bond density fluctuations exist on both the channel and particle surface, we next investigated the extent to which such variations in bond density could affect the stability of the particle-surface interface and hence account for the fluctuations we observe in the rolling adhesion experiments. We tested this using a series of flow-induced particle dissociation experiments, in which we systematically varied the bond density on the channel surface. A batch of 4.34 μm streptavidin-coated polystyrene particles was functionalized with a DNA duplex containing a sticky end of the sequence: 5'-Spacer-TTTT-ACTGGTCCTGCGGCG-3', hybridized to the spacer-C sequence, using the functionalization method described in the Materials & Methods section. Similarly, the channel surface was functionalized with a sticky-ended DNA duplex complementary to the one on the particles, of sequence: 5'-Spacer-TTTT-CGCCGCAGGACCAGT-3', hybridized to the spacer-C sequence. Consequently, shear-loaded slip bonds of length 15 nucleotides are formed between the particle and the channel surface. The bond density on the channel surface is varied by functionalizing with a mixture of the sticky ended DNA duplex (target DNA) and the spacer duplex. We then perform a particle dissociation experiment as follows: First, a batch of particles is introduced into the channel and the flow is turned off for 5 minutes to allow the particles to settle and form bonds with the channel surface. Next, the flow rate is increased up to a target shear rate within 1 second, causing the particles to dissociate from the channel surface. The particle dissociation was recorded over time using brightfield microscopy. We performed these experiments for three bond densities: 2.5%, 5% and 10% target DNA. At each bond density, we performed a series of measurements with varying shear rate. In each measurement, we observe a distribution of particle dissociation times. We determine the typical particle dissociation time τ by fitting the probability $p(t)$ that a particle survives up to a certain time t with a

stretched exponential function:

$$\begin{cases} p(t) = 1 & t < b \\ p(t) = exp\left[-\left(\frac{t-b}{\tau}\right)^{\alpha}\right] & t \geq b \end{cases} \tag{1}$$

Here, τ is the typical particle lifetime, α represents the stretched exponent and b denotes an offset that marks the time at which the flow rate is turned on ($t = 6$ s). A series of these stretched exponential fits for 2.5% Target DNA is shown in Supplementary fig. 2a. A plot of τ as a function of $\dot{\gamma}$ at varying percentages of target DNA reveals that small variations in bond density dramatically affects the mechanical stability of the particles-surface interface (Supplementary Fig. 2b). Hence, it is likely that the bond density fluctuations we observed in our fluorescence assay account substantially for the experimental fluctuations we observe.

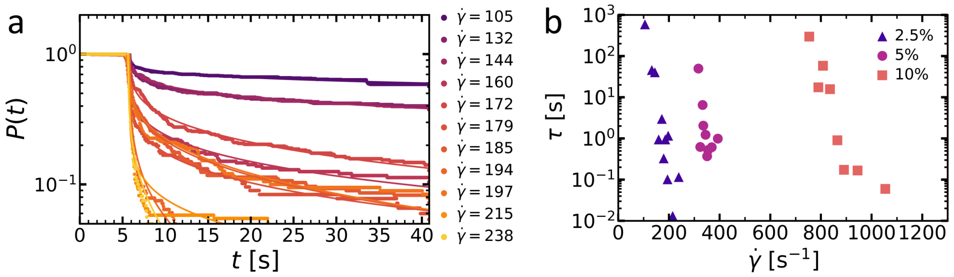

Supplementary Fig. 2. a: Particle survival probability $p(t)$ over t for a series of dissociation experiments at varying $\dot{\gamma}$ at 2.5% target DNA. Fits of eq. 1 are shown as solid lines. The flow rate was turned on at $t = 6$ s. b: Plots of τ as a function of $\dot{\gamma}$ at three % target DNA.

Finally, we investigated whether the experimental fluctuations could be caused by local shear rate variations across the width of the microfluidic channel. As the microfluidic channel is rectangular, we expect that the shear rate around the edges varies, because of a change in the flow velocity profile in these locations[5]. For this reason, we have recorded our rolling adhesion experiments in a smaller field-of-view in the center of the channel. To test whether some remnant of the varying flow velocity profile is still present in our field of view, we binned the particle dissociation times τ according to the particle positions across the width of the channel imaging window y, to generate the histograms shown in Supplementary Fig. 3. We find no clear trend indicating a larger dissociation time near the edges of the imaging window, which suggests that shear rate variations across the channel width do not play a role.

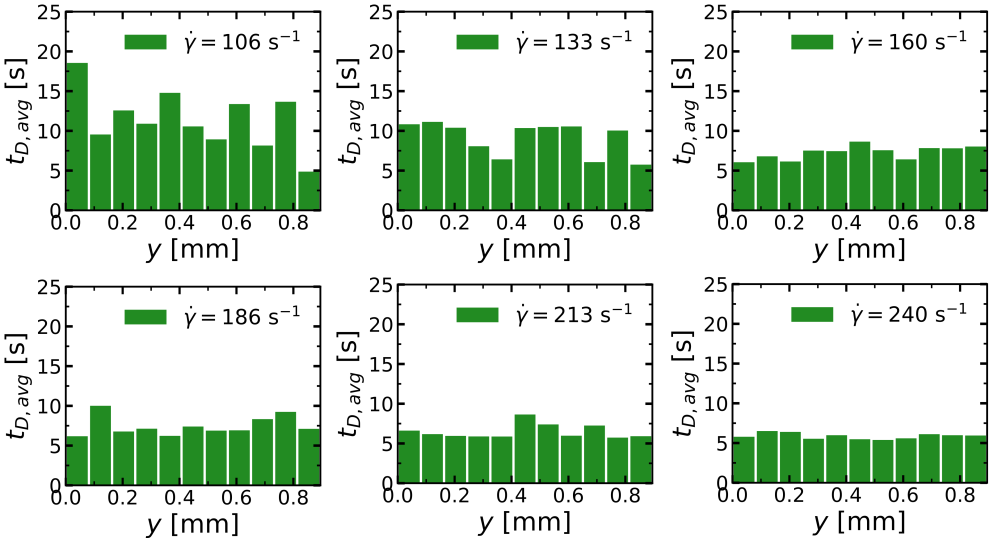

Supplementary Figure 3. Particle dissociation time (t_D) histograms binned according to the particle position across the width of the field of view (y-direction), recorded at varying $\dot{\gamma}$ at 2.5% target DNA.

Rolling adhesion simulation protocol

Rolling adhesion simulations were run using a custom-developed simulation program written in Python, adapted from a protocol we developed previously [6], the code of which is available on https://github.com/jorissprakel/DNACatchBond/. Chosen values for the simulation parameters are as follows:

Parameters	Description	Value	Unit	Source
ρ	Linker density on channel	350	$\#/\mu m^2$	estimate
a	Microparticle radius	0.68	μm	Spherotech inc.
L_K	Linker Kuhn length	56	nm	Manning, G.S.[7]
$k_{on,0}$	Linker formation rate	10ˆ4	s^{-1}	Estimate
**SB Parameters**				
$k_{off,0}$	Slip bond dissociation rate	$1{\cdot}10^{-1}$	s-1	Strunz et al.[8]
Δx	Mechanical susceptibility	1.26	nm	Strunz et al.[8]
L_{max}	Linker contour length	35.36	nm	Calculated[9, 10]
**CB Parameters**				
$k^1_{off,0}$	Weak state dissociation rate	$3.2{\cdot}10^{-2}$	s^{-1}	Strunz et al.[8]
$k^2_{off,0}$	Strong state dissociation rate	$1.0{\cdot}10^{-5}$	s^{-1}	Strunz et al.[8]
$k_{IC,0}$	Base interconversion rate	$3.2{\cdot}10^{-7}$	s^{-1}	Strunz et al.[8]
Δx^1	Weak state (1) susceptibility	1.3	nm	Strunz et al.[8]
Δx^2	Strong state (2) susceptibility	1.8	nm	Strunz et al.[8]
α^{IC}	Force localisation factor for IC	1		Estimate
L^1_{max}	contour length, weak state	35.36	nm	Calculated[9, 10]
L^2_{max}	contour length, strong state	57.72	nm	Calculated[9, 10]

All Bell-Evans parameters ($k^i_{off,0}$ and Δx^i), where calculated according to the models provided by Struntz et al. [8]. Contour lengths L_{max} in both the inactive and active states were calculated assuming a contour length of 0.34 nm per DNA base pair, and including two streptavidin units of size 5 nm[10] and two BSA proteins of size 5.5 nm[11]. A flow chart of the rolling adhesion simulation steps is shown below. Simulations are comprised of three stages: I. initiation, II. equilibration, and III the main simulation loop. Below, we explain each of these stages in detail.

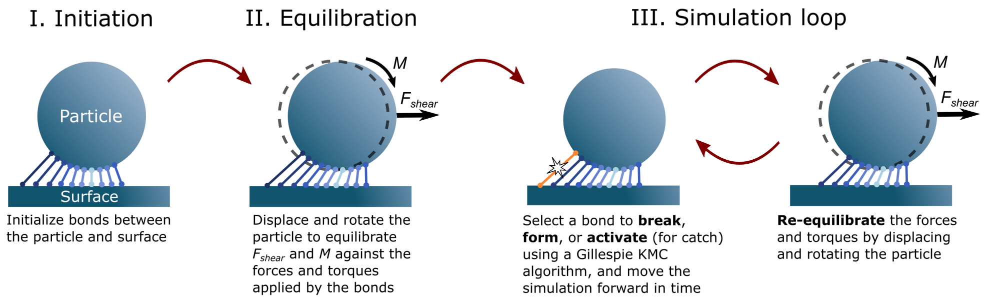

Supplementary fig. 4: Flowchart portraying the rolling adhesion simulation steps.

I. Initiation

To start a simulation, we place a particle in a 3D space, directly on top of a flat flow-cell surface. We then generate a square pattern of linker sites l_0 on top of the flow cell, such that the average density of linker sites equals $\rho = 2000\ \mu\text{m}^{-2}$. These linkers were then randomly displaced with a distance of 0.1x the distance between the linker sites to create a disordered pattern of linker sites. Next, we initiate slip/catch bonds between the microparticle and that on the flow cell as follows:

1. We calculate the distance r from the linker sites l_0 to the nearest position l_1 on the microparticle. These distances r indicate the bond lengths of the candidate bonds, assuming that the linker site density on the microparticle surface is large compared to the flow cell surface.
2. For each candidate bond, we use the the Worm-Like-Chain (WLC) force-extension model to determine the free energy change upon bond formation ΔG. This model provides an accurate approximation for DNA duplex extension [12].

$$\Delta G = \frac{2k_BT}{L_K}\left(\frac{1}{4}\frac{x^2}{1-x} + \frac{1}{2}x^2 - \frac{0.8}{3.15}x^{3.15}\right) \tag{2}$$

   Here, x=r/L_{max} describes the relative extension of a bond compared to the contour length L_{max}, and L_K is the Kuhn length. We did not include bonds with $x > 0.99$, as the standard WLC model is no longer valid in this regime.
3. Next, we connect a number of the linkers randomly using a pseudo-random number generator. For catch bond simulations, each bond is initialized in weak state 1, as this is the thermodynamically favorable state of the catch bond. To obey detailed balance, we select linkers to form according to:

$$K = \frac{k_{on,0}}{k_{off,0}} \cdot \exp[-\Delta G] \tag{3}$$

   Here, rate constants $k_{on,0}$ and $k_{off,0}$ indicate the linker formation and dissociation rates when no tension is applied. Through the Boltzmann weighing factor $\exp[-\Delta G]$, equation 3 ensures that highly stretched bonds are less likely to form than less stretched bonds.

II. Equilibration

After forming an initial number of linkers, we equilibrate the system. We first determine the force F_{shear} and torque M_{shear} applied to the microparticle at the current shear rate $\dot{\gamma}$, by interpolating between the values computed by Goldman et al. [13]. Using the WLC model [14], we then determine the tensional force on each bond:

$$F = \frac{\delta(\Delta G)}{\delta x} = \frac{2k_BT}{L_K}\left(\frac{1}{4}(1-x)^{-2} - \frac{1}{4} + x - 0.8x^{2.15}\right) \tag{4}$$

In the event that a bond extension exceeds $x > 0.99$, its tensional force is set to to $F(x = 0.989)$. This prevents computational instabilities arising from the invalidity of

the WLC model at these large extensions. From the applied forces, the total torque M_y in the y-direction are then calculated as follows:

$$M_y = \sum_i ((z_i - h) \cdot F_{x,i} - x_i \cdot F_{z,i}) \tag{5}$$

In eq. 5, the horizontal and vertical components of the linker force of each linker i are given as $F_{x,i}$ and $F_{z,i}$, z_i denotes the height of linker position l_1 on the microparticle surface, and the microparticle's center-of-mass is indicated as h. Having computed the forces and torques applied to the microparticle through the applied shear and the bonds, we equilibrate the system using a gradient descent algorithm, until the forces and torques are balanced. During the equilibration, we allow two degrees of freedom of the microparticle: translation along the x-direction and rotation around the y-axis. Other degrees of freedom are neglected for computational efficiency.

III. Running the simulations

After equilibration, we start the following simulation loop, consisting of two steps: 1) We randomly select one linker to to activate (only for catch bonds), dissociate or form, using an event-driven kinetic Monte Carlo algorithm (KMC) [15], and we move the simulation forward in time. 2) We equilibrate the system, using the equilibration algorithm described in the previous section. By repeating steps 1 and 2 we simulate the rolling microparticle over time.

Each kinetic Monte Carlo step is comprised of the following steps:

1. We first compute the rate constants for all possible reaction steps the system can make: bond dissociation, bond formation and catch bond activation. Here, the protocols for slip bond simulations and catch bond simulations are somewhat different:

   For slip bonds, there are two relevant rate constants: a formation rate constant k_{on} for each unbound linker and a dissociation rate constant k_{off} for each bound linker. We model the force-dependence of k_{off} using Bell-type mechano-enhanced kinetics [16]:

   $$k_{off} = k_{off,0} \cdot \exp\left[\frac{F\Delta x}{k_B T}\right] \tag{6}$$

   with $k_{off,0}$ the rate constant at $F = 0$ and Δx the mechanical susceptibility to force. For each bound linker, we compute k_{off} using the linker extension force determined by eq. 4. The formation rate constant k_{on} is also extension-dependent, as highly stressed linkers are less likely to form. k_{on} is computed as:

   $$k_{on} = k_{on,0} \cdot \exp\left[\frac{F\Delta x - \Delta G}{k_B T}\right] \tag{7}$$

with $k_{on,0}$ the rate constant at $F = 0$. Equation 7 is chosen such that $K = \frac{k_{on}}{k_{off}} = \frac{k_{on,0}}{k_{off,0}} \exp[-\Delta G]$, which ensures that K is determined by ΔG for every extension force F.

For catch bonds, each linker can be either unbound, bound in weak state 1 or bound in strong state 2. Linkers from these states can dissociate through rate constants k^1_{off} and k^2_{off}, which are modelled through eq. 8, using the $k^{1/2}_{off,0}$ and Δx^{1-2} parameters described in table . As for slip bonds, the formation rate constant k_{on} is modeled through equation 7. In these simulations, we assume that bonds always form in the weak state 1, and never in the strong state 2, as state 1 is the more stable state in absence of tension. Interconversion of bonds from state 1 to state 2 is accounted for by rate constant k_{IC}, which is modelled through an adaptation of equation 8:

$$k_{IC} = k_{IC,0} \cdot \exp\left[\frac{F \Delta x^{IC} \alpha^{IC}}{k_B T}\right] \tag{8}$$

here, the force localisation factor α^{IC}, accounts for the increased mechanical susceptibility of the hairpin due to the zipper-type loading geometry.Finally, we assume that relaxation from strong state 2 to weak state 1 can be neglected, since the bonds remain under tension until they dissociate.

2. Having computed the rate constants of all potential transitions, we next select one transition randomly, using the rate constants as weights. This makes faster transitions more likely to occur [15]. Using the protocol described by Gillespie et al., we determine the transition time Δt after each KMC step, allowing us to simulate the development of the interface over time. In this protocol, we assume that the force and torque equilibration is fast compared to the KMC step, allowing us to neglect relaxations caused by dissipation. We expect this is a reasonable assumption, as there are typically many bonds between the microparticle and the flow cell, so that individual dissociated or formed bonds have a small impact on the system as a whole.

For both slip bonds and catch bonds, a series of 100 simulations of $1 \cdot 10^6$ KMC steps were run at each shear rate. Every 100 simulation steps the simulation time t, the microparticle's location x_P and rotation angle θ, and the number of bonds in the weak and strong states are stored. To compute the catch bond activation heatmap, an additional series of 20 simulations of $1 \cdot 10^6$ KMC steps was run at $\dot{\gamma} = 560$ s^{-1}, during which the position, force and state of each catch bond linker was stored every 2500 steps.

List of supplementary videos

- **SupplementaryVideo_1.avi.** Render of an oxDNA MD simulation showing a catch bond interconverting from state 1 to state 2 under 30 pN of overall tension. Under tension, the hairpin opens quickly, which is then followed by a closing of the latch to yield state 2. Simulation consisted of $1.6 \cdot 10^9$ MD steps in total, and a snapshot is shown every $2 \cdot 10^6$ simulation steps.
- **SupplementaryVideo_2.avi.** Render of an oxDNA MD simulation showing a catch bond relaxing from state 2 to state 1 in absence of tension. Initially, the toe-hold forms rapidly, followed by an internal strand exchange that displaces the complementary strand and reforms the hairpin to yield state 1. Simulation consisted of $1.6 \cdot 10^9$ MD steps in total, and a snapshot is shown every $2 \cdot 10^6$ simulation steps.
- **SupplementaryVideo_3.avi.** Close-up of a typical rolling adhesion experiment using slip bonds (SB-1 and SB-2) performed at $\dot{\gamma} = 400\ s^{-1}$ (left) and $\dot{\gamma} = 667\ s^{-1}$ (right). Particle traces are shown as yellow lines, and tracked particles are color-coded based on whether they are in a rolling regime (green) or stopping regime (red). A clear increase in rolling velocity is observed at increasing $\dot{\gamma}$.
- **SupplementaryVideo_4.avi.** Close-up of a typical rolling adhesion experiment using catch bonds (CB-HS and CB-CS) performed at $\dot{\gamma} = 400\ s^{-1}$ (left) and $\dot{\gamma} = 667\ s^{-1}$ (right). Particle traces are shown as yellow lines, and tracked particles are color-coded based on whether they are in a rolling regime (green) or stopping regime (red). At $\dot{\gamma} = 400\ s^{-1}$ a larger fraction of particles display rolling behavor than at the higher shear rate of $\dot{\gamma} = 667\ s^{-1}$.

Particle numbers

Table S3. Overview of the number of particles N measured for every $\dot{\gamma}$ datapoint in figure 2c, for the slip bond and the catch bond ($N_{Latch} = 7$) data.

Slip		Catch bond	
$\dot{\gamma}[s^{-1}]$	N	$\dot{\gamma}[s^{-1}]$	N
200	157	200	146
267	380	300	168
333	508	333	216
400	169	367	212
467	831	400	305
533	344	433	226
600	1200	467	363
667	698	500	349
733	2157	533	240
800	1027	567	273
867	1508	600	385
933	788	667	402
1000	1167		
1067	712		
1133	961		
1200	649		

Table S4. Overview of the number of particles N measured for every $\dot{\gamma}$ datapoint in figures 2e, and 2f, for the slip bond and the catch bond ($N_{Latch} = 7$) data.

Slip bond		Catch bond	
$\dot{\gamma}[s^{-1}]$	N	$\dot{\gamma}[s^{-1}]$	N
200	94	200	74
266.67	163	300	97
333.33	409	333.33	97
400	125	366.67	91
466.67	570	400	151
533.33	239	433.33	107
600	925	466.67	141
666.67	503	500	157
733.33	1359	533.33	148
800	597	566.67	127
866.67	970	600	142
933.33	473	666.67	121
1000	737		
1066.67	488		
1133.33	538		
1200	372		

Table S5. Overview of the number of particles N measured for every $\dot{\gamma}$ datapoint in figure 4a, for the slip bond and each N_{Latch} variation.

Slip		Nlatch = 2		Nlatch = 3		Nlatch = 4		Nlatch = 5		Catch bond	
$\dot{\gamma}[s^{-1}]$	N	$\dot{\gamma}[s^{-1}]$	N	$\dot{\gamma}[s^{-1}]$	N	$\dot{\gamma}[s^{-1}]$	N	$\dot{\gamma}[s^{-1}]$	N	$\dot{\gamma}[s^{-1}]$	N
200	94	133.33	190	266.67	44	133.33	45	133.33	153	200	74
266.67	163	266.67	234	533.33	126	266.67	83	266.67	194	300	97
333.33	409	400	381	800	512	400	83	400	238	333.33	97
400	125	533.33	438	1066.67	938	533.33	182	533.33	130	366.67	91
466.67	570	666.67	646			666.67	175	666.67	65	400	151
533.33	239	800	521			800	206	800	71	433.33	107
600	925	933.33	810			933.33	246	933.33	67	466.67	141
666.67	503	1066.67	994			1066.67	254	1066.67	79	500	157
733.33	1359	1200	156			1200	341	1200	82	533.33	148
800	597									566.67	127
866.67	970									600	142
933.33	473									666.67	121
1000	737										
1066.67	488										
1133.33	538										
1200	372										

Table S6. Overview of the number of particles N measured for every $\dot{\gamma}$ datapoint in figure 4b, for the slip bond and each N_{Latch} variation.

**Slip**		**Nlatch = 2**		**Nlatch = 3**		**Nlatch = 4**		**Nlatch = 5**		**Catch bond**	
$\dot{\gamma}$[s^{-1}]	N	$\dot{\gamma}$[s^{-1}]	N	$\dot{\gamma}$[s^{-1}]	N	$\dot{\gamma}$[s^{-1}]	N	$\dot{\gamma}$[s^{-1}]	N	$\dot{\gamma}$[s^{-1}]	N
200	157	133	444	267	232	133	233	133	288	200	146
267	380	267	530	533	731	267	288	267	475	300	168
333	508	400	910	800	1295	400	156	400	432	333	216
400	169	533	961	1067	1725	533	329	533	519	367	212
467	831	667	1301			667	365	667	101	400	305
533	344	800	1291			800	451	800	148	433	226
600	1200	933	1779			933	538	933	201	467	363
667	698	1067	1964			1067	615	1067	269	500	349
733	2157	1200	232			1200	790	1200	302	533	240
800	1027									567	273
867	1508									600	385
933	788									667	402
1000	1167										
1067	712										
1133	961										
1200	649										

References

[1] Suma, A. *et al.* Tacoxdna: A user-friendly web server for simulations of complex dna structures, from single strands to origami. *J. Comput. Chem.* **40**, 2586–2595 (2019).

[2] Dimura, M. *et al.* Quantitative fret studies and integrative modeling unravel the structure and dynamics of biomolecular systems. *Curr. Opin. Struct. Biol.* **40**, 163–185 (2016).

[3] Šulc, P. *et al.* Sequence-dependent thermodynamics of a coarse-grained dna model. *J. Chem. Phys.* **137**, 135101 (2012).

[4] Bohlin, J. *et al.* Design and simulation of dna, rna and hybrid protein–nucleic acid nanostructures with oxview. *Nat. Protoc.* **17**, 1762–1788 (2022).

[5] Figueroa-Morales, N. *et al.* Living on the edge: transfer and traffic of e. coli in a confined flow. *Soft Matter* **11**, 6284–6293 (2015). URL http://dx.doi.org/10.1039/C5SM00939A.

[6] van Galen, M. *et al.* Rapid molecular mechanotyping with microfluidic force spectroscopy. *bioRxiv* 2023–02 (2023).

[7] Manning, G. S. The persistence length of dna is reached from the persistence length of its null isomer through an internal electrostatic stretching force. *Biophys. J.* **91**, 3607–3616 (2006). URL https://www.sciencedirect.com/science/article/pii/S0006349506720732.

[8] Strunz, T., Oroszlan, K., Schafer, R. & Guntherodt, H. Dynamic force spectroscopy of single dna molecules. *Proc. Natl. Acad. Sci. U.S.A.* **96**, 11277–11282 (1999).

[9] Neish, C. S., Martin, I. L., Henderson, R. M. & Edwardson, J. M. Direct visualization of ligand-protein interactions using atomic force microscopy. *Br. J. Pharmacol.* **135**, 1943–1950 (2002). URL https://bpspubs.onlinelibrary.wiley.com/doi/abs/10.1038/sj.bjp.0704660.

[10] González Flecha, F. L. & Levi, V. Determination of the molecular size of bsa by fluorescence anisotropy. *Biochem. Mol. Biol. Educ.* **31**, 319–322 (2003). URL https://iubmb.onlinelibrary.wiley.com/doi/abs/10.1002/bmb.2003.494031050261.

[11] Kubiak-Ossowska, K., Tokarczyk, K., Jachimska, B. & Mulheran, P. A. Bovine serum albumin adsorption at a silica surface explored by simulation and experiment. *The Journal of Physical Chemistry B* **121**, 3975–3986 (2017).

[12] Williams, M. C. & Rouzina, I. Force spectroscopy of single dna and rna molecules. *Curr. Opin. Struct. Biol.* **12**, 330–336 (2002).

[13] Goldman, A., Cox, R. & Brenner, H. Slow viscous motion of a sphere parallel to a plane wall .2. couette flow. *Chem. Eng. Sci.* **22**, 653–660 (1967).

[14] Petrosyan, R. Improved approximations for some polymer extension models. *Rheol. Acta* **56**, 21–26 (2017).

[15] Gillespie, D. T. A general method for numerically simulating the stochastic time evolution of coupled chemical reactions. *J. Comput. Phys.* **22**, 403–434 (1976).

[16] Bell, G. Models for specific adhesion of cells to cells. *Science* **200**, 618–627 (1978).

Supplementary Figures

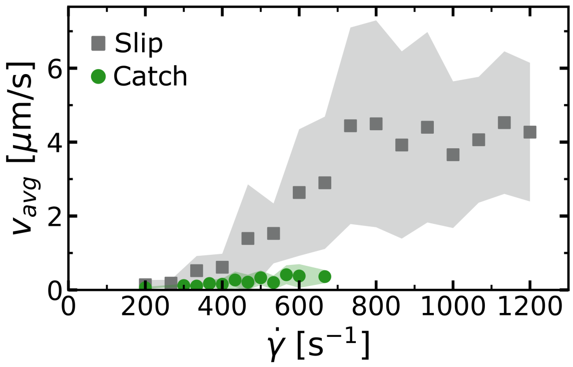

Supplementary figure 5. Overall average velocity v_{avg} for slip bonded (gray) and catch bonded (green) particles under increasing $\dot{\gamma}$. v_{avg} is the average velocity across the entire particle trajectory, including both the rolling and stopping regimes. Only particles that displayed some degree of rolling (minimal displacement $> 1\ \mu m$) were included in the analysis.

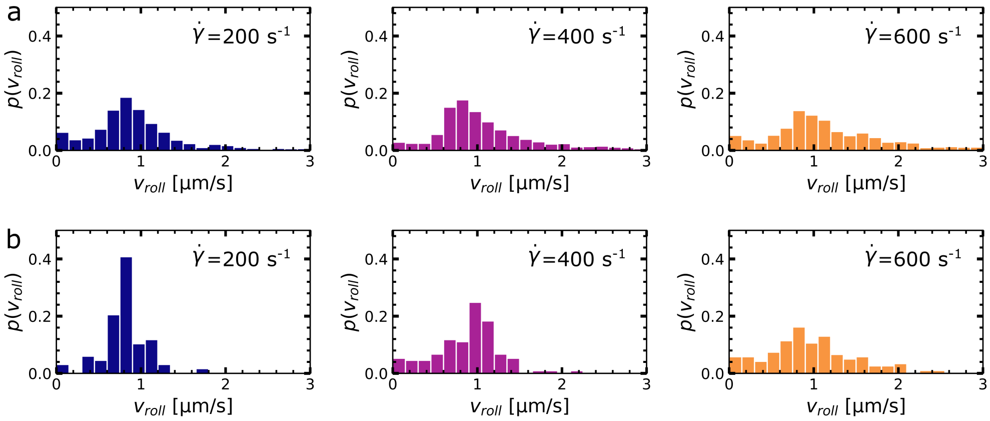

Supplementary figure 6. v_{roll} histograms for catch bonded particles at varying $\dot{\gamma}$, averaged by (a) roll segment and (b) particle trajectory. v_{roll} histograms in (a) contain substantial populations at large v_{roll}. These populations are reduced when the intrinsic fluctuations are averaged out by particle trajectory in (b).

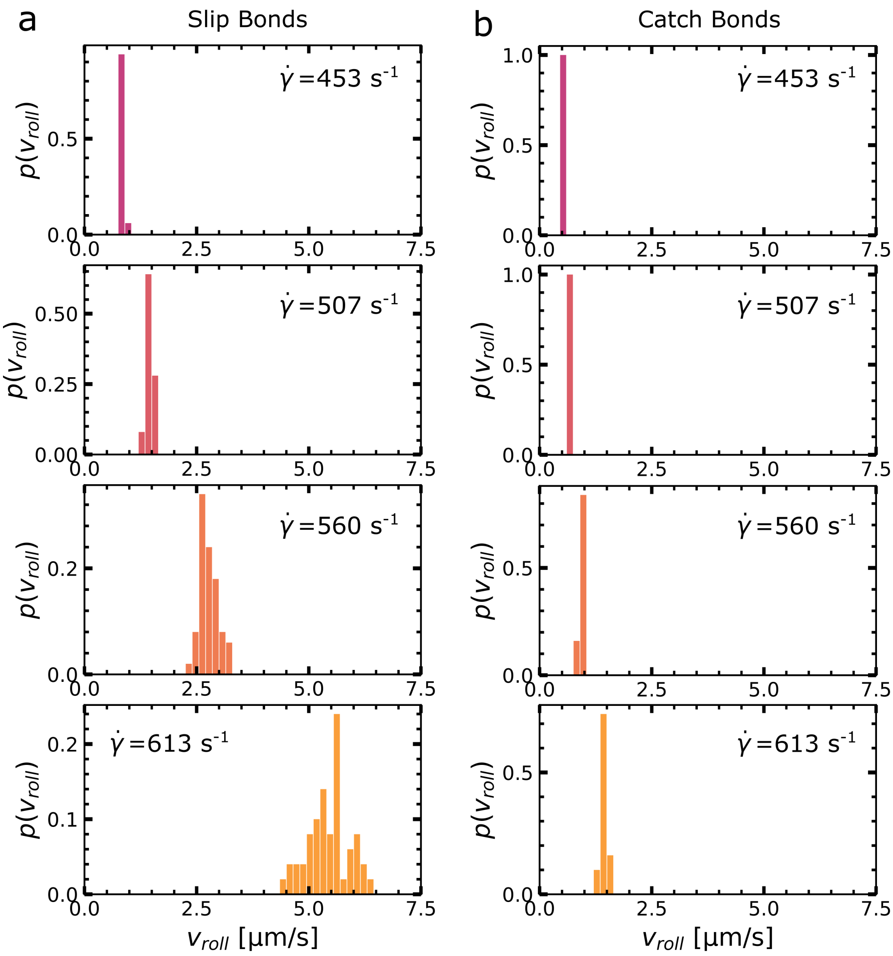

Supplementary figure 7. *Simulated* v_{roll} histograms for particles bound by (a) slip bonds and (b) catch bonds under increasing $\dot{\gamma}$.

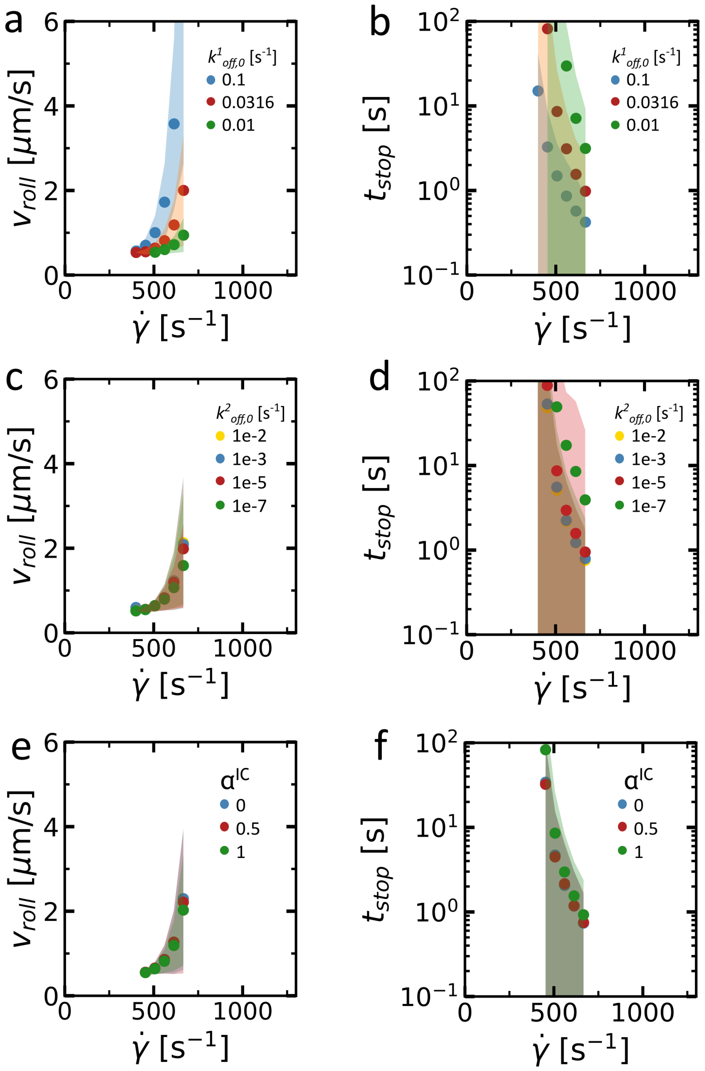

Supplementary figure 8. Parameter space exploration in the rolling adhesion simulations, varying (a,b): the weak state base dissociation rate $k^1_{off,0}$, (c,d): the strong state base dissociation rate $k^2_{off,0}$, (d,e): the stress localisation factor for interconversion α^{IC}. 50 particles were simulated for each datapoint. All non-varied parameters were set to their standard value described in the simulation protocol in the SI.

Schematic representations each DNA construct in the weak and strong states.

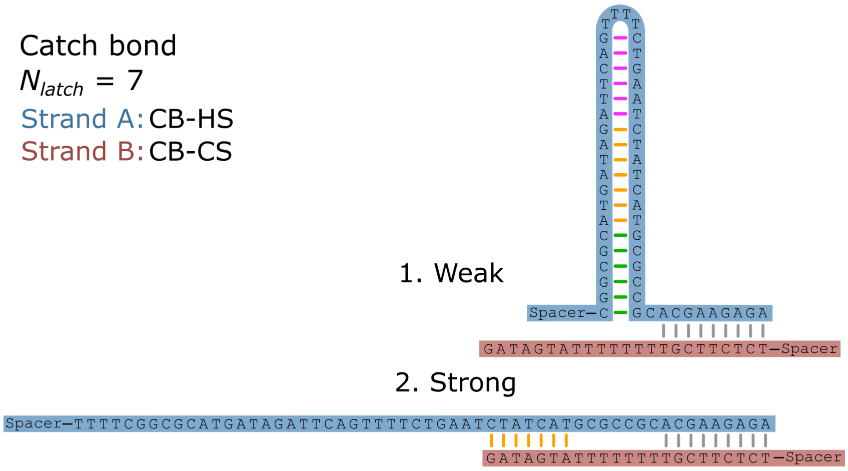

Supplementary figure 9. Detailed sketch of the main catch bond construct $N_{Latch} = 7$ in states 1. weak and 2. strong.

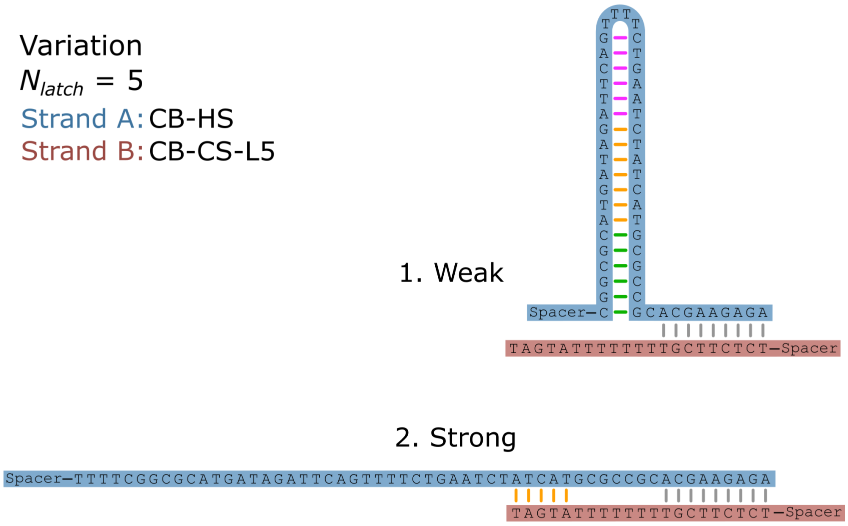

Supplementary figure 10. Detailed sketch of catch bond variation $N_{Latch} = 5$ in states 1. weak and 2. strong.

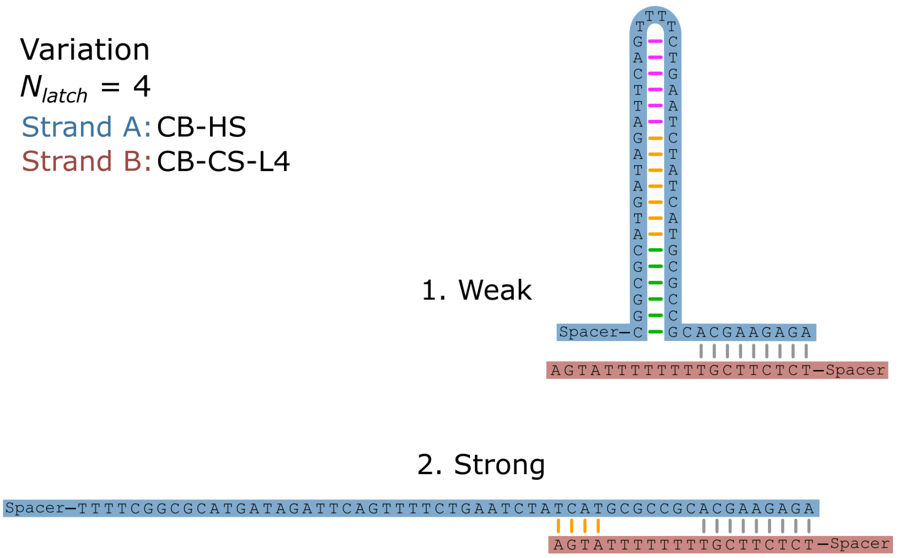

Supplementary figure 11. Detailed sketch of catch bond variation $N_{Latch} = 4$ in states 1. weak and 2. strong.

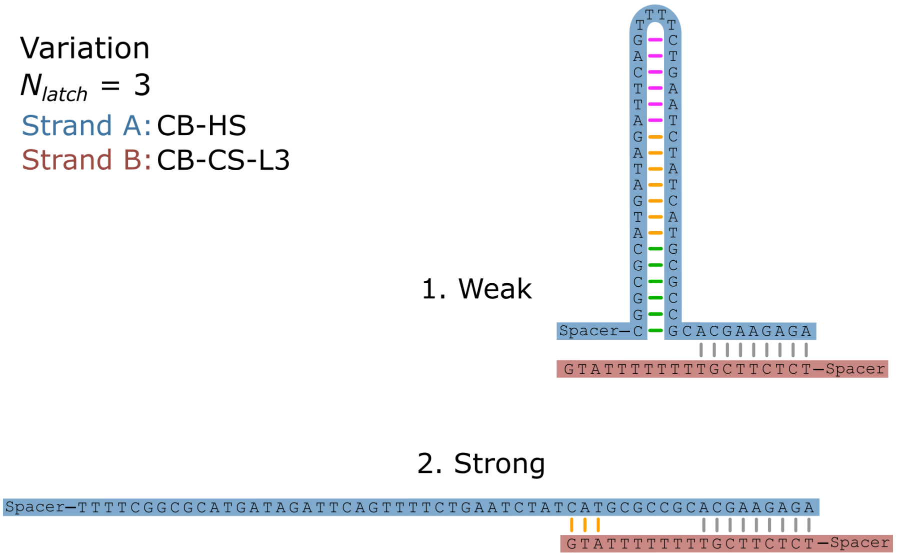

Supplementary figure 12. Detailed sketch of catch bond variation $N_{Latch} = 3$ in states 1. weak and 2. strong.

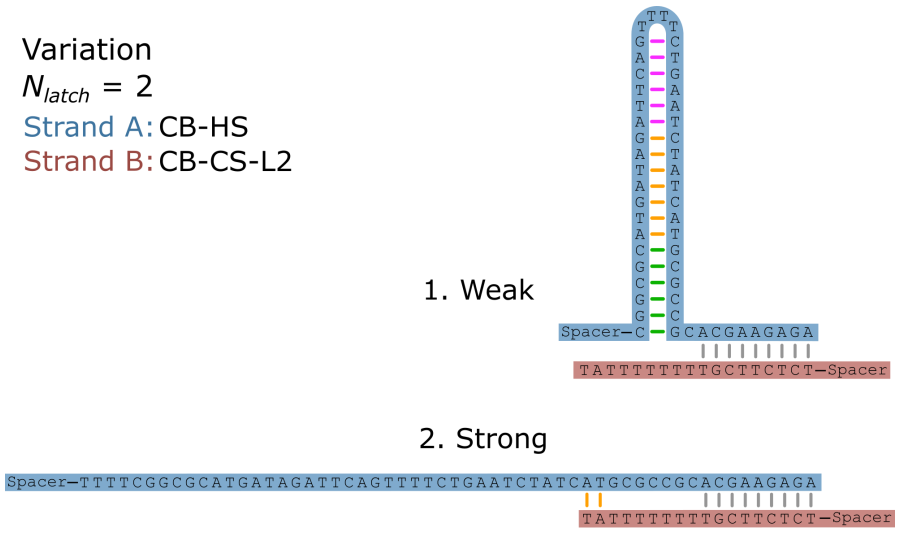

Supplementary figure 13. Detailed sketch of catch bond variation $N_{Latch} = 2$ in states 1. weak and 2. strong.

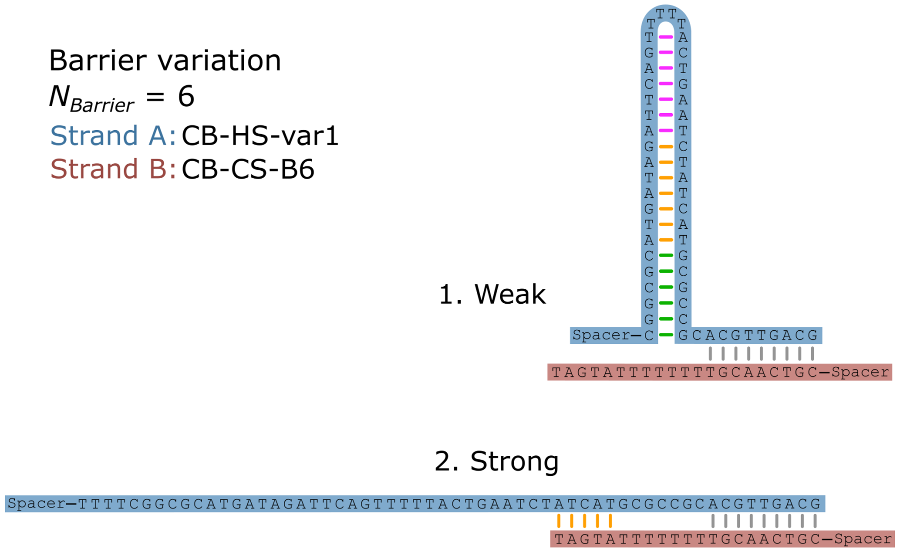

Supplementary figure 14. Detailed sketch of barrier sequence variations $N_{Barrier} = 6$ in states 1. weak and 2. strong.

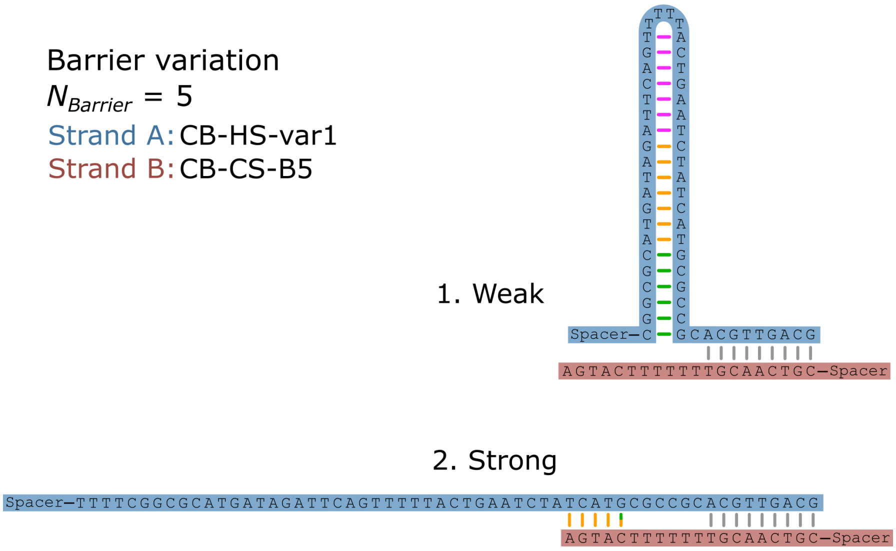

Supplementary figure 15. Detailed sketch of barrier sequence variations $N_{Barrier}$ = 5 in states 1. weak and 2. strong.

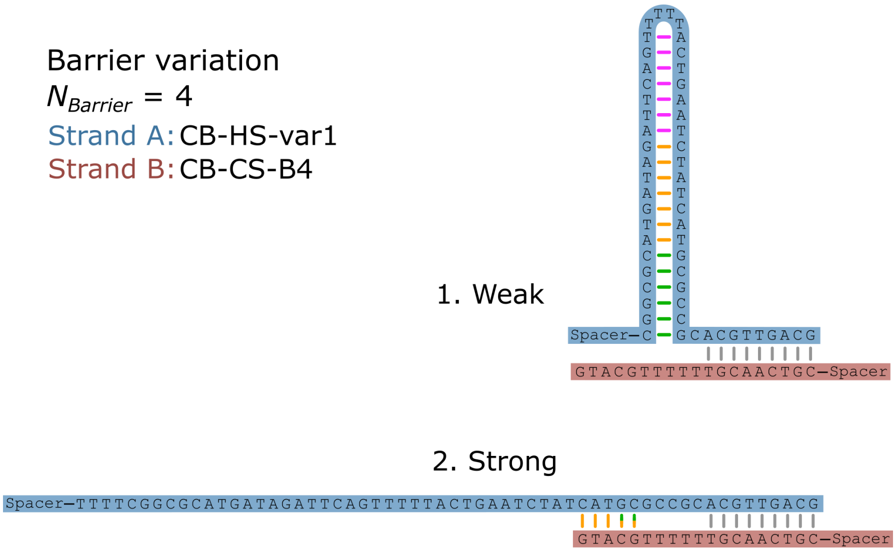

Supplementary figure 16. Detailed sketch of barrier sequence variations $N_{Barrier}$ = 4 in states 1. weak and 2. strong.

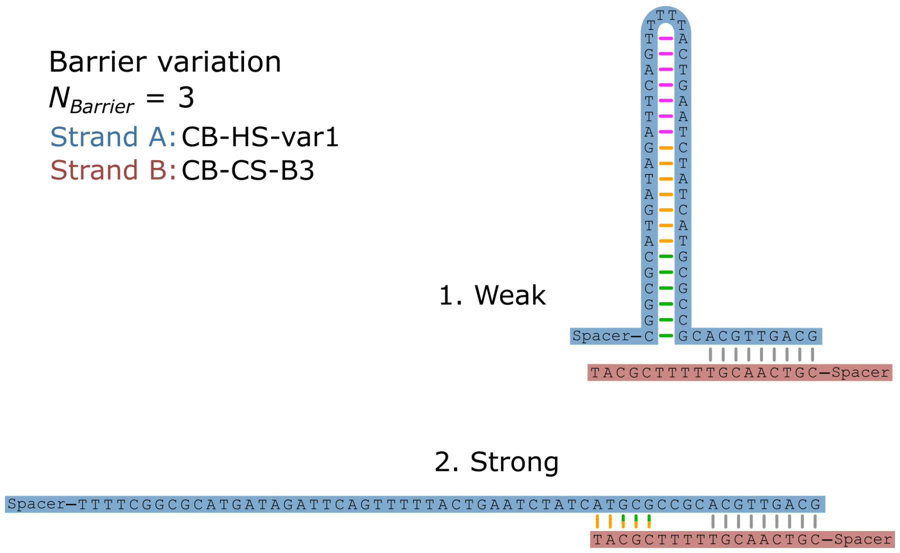

Supplementary figure 17. Detailed sketch of barrier sequence variations $N_{Barrier} = 3$ in states 1. weak and 2. strong.

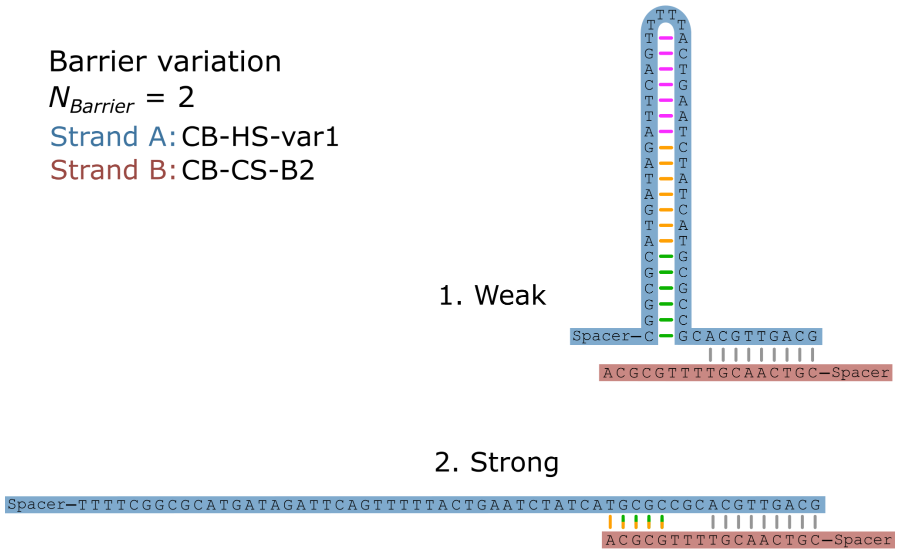

Supplementary figure 18. Detailed sketch of barrier sequence variations $N_{Barrier} = 2$ in states 1. weak and 2. strong.

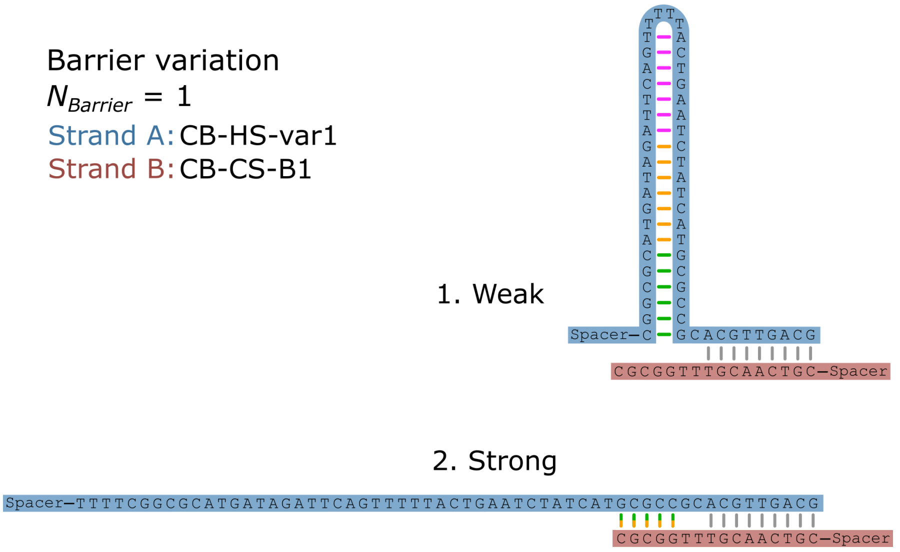

Supplementary figure 19. Detailed sketch of barrier sequence variations $N_{Barrier} = 1$ in states 1. weak and 2. strong.

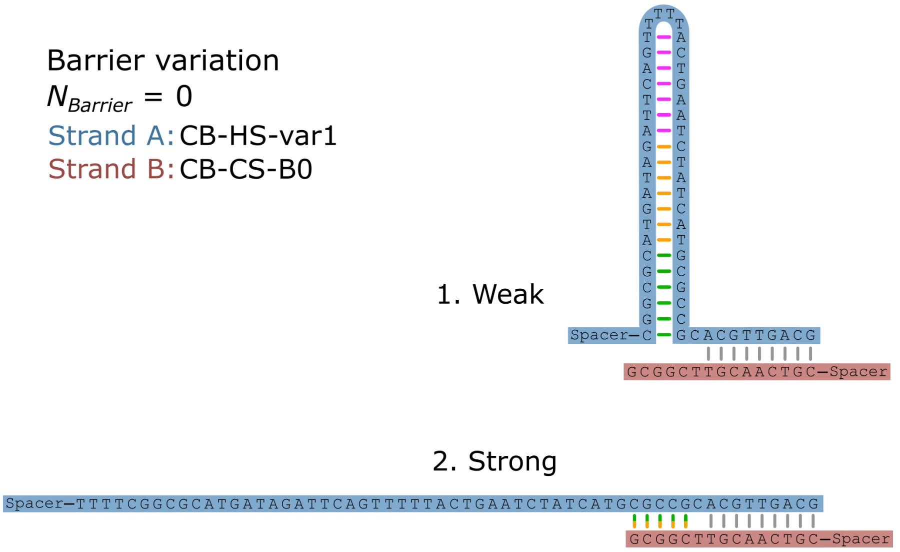

Supplementary figure 20. Detailed sketch of barrier sequence variations $N_{Barrier} = 0$ in states 1. weak and 2. strong.
